# Three autoinducer molecules act in concert to control virulence gene expression in *Vibrio cholerae*

**DOI:** 10.1093/nar/gky1320

**Published:** 2019-01-15

**Authors:** Roman Herzog, Nikolai Peschek, Kathrin S Fröhlich, Kilian Schumacher, Kai Papenfort

**Affiliations:** 1 Faculty of Biology I, Department of Microbiology, Ludwig-Maximilians-University of Munich, 82152 Martinsried, Germany; 2 Munich Center for Integrated Protein Science (CIPSM), Germany

## Abstract

Bacteria use quorum sensing to monitor cell density and coordinate group behaviours. In *Vibrio cholerae*, the causative agent of the diarrheal disease cholera, quorum sensing is connected to virulence gene expression via the two autoinducer molecules, AI-2 and CAI-1. Both autoinducers share one signal transduction pathway to control the production of AphA, a key transcriptional activator of biofilm formation and virulence genes. In this study, we demonstrate that the recently identified autoinducer, DPO, also controls AphA production in *V. cholerae*. DPO, functioning through the transcription factor VqmA and the VqmR small RNA, reduces AphA levels at the post-transcriptional level and consequently inhibits virulence gene expression. VqmR-mediated repression of AphA provides an important link between the AI-2/CAI-1 and DPO-dependent quorum sensing pathways in *V. cholerae*. Transcriptome analyses comparing the effect of single autoinducers versus autoinducer combinations show that quorum sensing controls the expression of ∼400 genes in *V. cholerae* and that all three autoinducers are required for a full quorum sensing response. Together, our data provide a global view on autoinducer interplay in *V. cholerae* and highlight the importance of RNA-based gene control for collective functions in this major human pathogen.

## INTRODUCTION

To efficiently interact with their environment, bacteria often work in groups to solve complex tasks. Coordination of collective functions requires communication among the members of the group, a process commonly referred to as quorum sensing (QS) (1,2). QS involves the production, release, and subsequent detection of extracellular small molecules called autoinducers.

**Figure 1.**
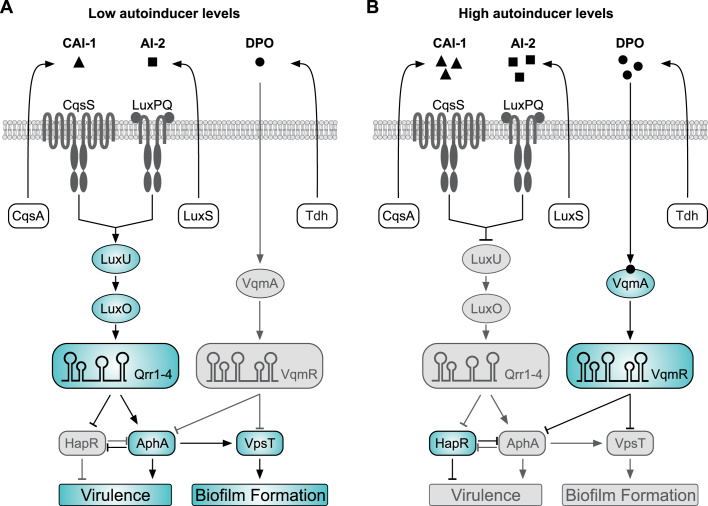
Quorum sensing in *V. cholerae* is controlled by three autoinducer molecules. The CAI-1 and AI-2 autoinducers are produced by CqsA and LuxS and detected by the membrane-bound CqsS and LuxPQ receptors, respectively. The DPO autoinducer derives from threonine catabolism, and requires the Tdh (threonine dehydrogenase) enzyme. DPO is released into the environment and binds to and activates the VqmA receptor. (**A**) At low autoinducer concentrations, CqsS and LuxPQ act as kinases to phosphorylate LuxU. LuxU-P transfers the phosphate to LuxO, and LuxO-P induces the expression of the Qrr1–4 sRNAs. The Qrr sRNAs act post-transcriptionally to repress *hapR* and activate *aphA*, promoting virulence gene expression and biofilm formation. AphA also activates the transcription of *vpsT*. (**B**) At high autoinducer concentrations, binding of CAI-1 and AI-2 to CqsS and LuxPQ, respectively, converts the receptors to phosphatases, which reduces LuxO-P levels and inhibits *qrr1–4* expression. Under these conditions, *aphA* is repressed and *hapR* is activated. The VqmA-DPO complex induces the transcription of the VqmR sRNA. VqmR inhibits biofilm formation by repressing VpsT and virulence gene expression by inhibiting AphA. In addition, HapR and AphA antagonize each other at the transcriptional level. Active factors are highlighted in blue, inactive (repressed) factors are shown in gray.

In *Vibrio cholerae*, the causative agent of cholera disease, QS is intimately linked to several collective functions, including biofilm formation (3), type VI secretion (4,5), competence (6,7), phage resistance (8) and virulence gene expression (9). The canonical QS pathway of *V. cholerae* (Figure 1A and B) involves the two autoinducer molecules, CAI-1 ((*S*)-3-hydroxytridecan-4-one) and AI-2 ((2*S*,4*S*)-2-methyl-2,3,3,4-tetrahydroxytetrahydrofuran borate). CAI-1 and AI-2 are synthesized by the CqsA and LuxS enzymes and accumulate to concentrations of ∼0.3 μM (CAI-1) and ∼1–2 μM (AI-2) in cell-free supernatants of *Vibrio* species (10,11). Their cognate receptors are the membrane-bound proteins CqsS and LuxPQ, respectively (11–16). Both, CqsS and LuxPQ channel phosphate to the phospho-transfer protein LuxU, which transfers the phosphate to the response regulator LuxO (17). Phosphorylated LuxO together with the alternative sigma factor σ^N^ activates the expression of genes encoding four homologous regulatory small RNAs (sRNAs), called Qrr1-4 (18). The Qrr sRNAs act at the heart of the two QS systems by reciprocally controlling the production of the transcriptional regulators HapR and AphA, which regulate biofilm formation and virulence of *V. cholerae* (19). Importantly, the CqsS and LuxPQ receptors act as kinases in the absence of AI-2 and CAI-1, but convert to phosphatases when the autoinducers are present (20). Thus, expression of the Qrr sRNAs is repressed by AI-2 and CAI-1 (Figure 1B). In addition, two other receptor proteins, CqsR and VpsS, have been reported to channel information through LuxO, indicating the existence of at least four sensory inputs for this pathway (21).

Recently, we discovered another QS system operating in *V. cholerae* (Figure 1 and (22)). In contrast to CAI-1 and AI-2, this system does not require LuxU, LuxO or the Qrr sRNAs, but rather relies on the catabolic degradation of L-threonine by threonine dehydrogenase (encoded by *tdh*) and the concomitant synthesis of another autoinducer, called DPO (3,5-dimethylpyrazin-2-ol). DPO is sensed by VqmA, a cytoplasmic LuxR-type transcriptional regulator, which induces the transcription of the VqmR sRNA. VqmR belongs to the ubiquitous class of Hfq-associated regulatory RNAs (23) and we have shown that VqmR inhibits multiple *trans*-encoded target genes through direct base-pairing with their respective mRNAs (24). The target spectrum of VqmR also includes the transcript encoding VpsT, a key activator of biofilm formation in *V. cholerae* (24,25). Consequently, DPO, by acting through VqmA and VqmR, inhibits biofilm formation in *V. cholerae* (22).

Biofilm formation and pathogenicity are closely connected in *V. cholerae* (26,27). During the initial phases of infection, biofilms allow *V. cholerae* to survive the acidic environment of the stomach (28) and intravital microscopy revealed the formation of biofilms in the small intestines of infected mice (29). Therefore, perhaps not surprisingly, biofilm formation and intestinal colonization share a large set of co-regulated genes in *V. cholerae*. The two transcription factors, HapR and AphA, which are also regulated by QS (Figure 1), have overarching roles in both processes as they control the genes for biofilm and virulence regulation in an opposite manner (19). Specifically, AphA, together with another transcriptional regulator, called AphB (30), activates the production of the toxin-co-regulated pilus (TCP) and the cholera toxin (CTX). Both, TCP and CTX are necessary for infections in humans (31). Likewise, AphA activates VpsT production, which enhances biofilm formation (32). HapR antagonizes these functions by inhibiting the production of AphA and VpsT, as well as several other genes related to biofilm formation and virulence gene expression (33). Of note, HapR also controls Type VI secretion in *V. cholerae* (5), a process which has recently been reported to drive interspecies competition during host colonization (34,35).

In this study, we used RNA-sequencing to identify additional target mRNAs of VqmR in *V. cholerae*. Our analysis revealed five previously unknown target transcripts, including the *aphA* mRNA. We show that VqmR inhibits AphA production by interacting with the ribosome binding site (RBS) of the corresponding mRNA and that base-pairing involves the Rho-independent terminator sequence of VqmR. VqmR-mediated repression of AphA is stimulated by DPO and results in reduced virulence gene expression. Reduction of AphA levels by DPO connects the two QS pathways of *V. cholerae* at a critical regulatory node and suggests a coactive role in gene regulation. Indeed, global RNA-sequencing analysis of autoinducer-treated cells shows that QS controls more than 400 genes in *V. cholerae* and that AI-2, CAI-1 and DPO work together to control biofilm formation, virulence gene expression, and other collective functions in this major human pathogen.

## MATERIALS AND METHODS

### Strains, plasmids and growth conditions

Strains are listed in Supplementary Table S2. *V. cholerae* and *E. coli* were grown aerobically in LB or M9 minimal medium (0.4% glucose) at 37°C. Antibiotics were used at the following concentrations: 50 U ml^−1^ polymyxin B, 100 μg ml^−1^ ampicillin, 50 μg ml^−1^ kanamycin, 5000 μg ml^−1^ streptomycin, and 20 μg ml^−1^ chloramphenicol. Experiments involving AKI growth conditions were performed following previously published protocols (36).

### Oligonucleotides and plasmids

Plasmids and DNA oligonucleotides are listed in Supplementary Tables S3 and S4, respectively. Details on plasmid construction are provided in the Supplementary Methods section.

### Northern Blot analysis

Total RNA was prepared and transferred as previously described (37). Membranes were hybridized in Roti-Hybri-Quick buffer (Roth) at 42°C with [^32^P] end-labelled DNA oligonucleotides, or 63°C when using riboprobes. Signals were visualized using a Typhoon phosphorimager (Amersham) and band intensities were quantified using the GelQuant software (biochemlabsolutions). Oligonucleotides for Northern Blot analyses are provided in Supplementary Table S4.

### Western Blot analysis and fluorescence assays

Western Blot analyses of GFP and FLAG fusion proteins followed previously published protocols (38). Signals were visualized using a Fusion FX EDGE imager (Vilber) and band intensities were quantified using the BIO-1D software (Vilber). Fluorescence assay of *V. cholerae* and *E. coli* strains were performed as previously described (22,37).

### Preparation of secreted protein fractions

The cell densities (OD_600_) of AKI cultures were determined after 16h of continuous shaking. Two milliliter of each culture were centrifuged at 13 000 rpm for 30 min at 4°C and 1.6 ml of the supernatants were transferred to a new reaction tube. To precipitate secreted proteins, 0.4 ml of 25% ice-cold trichloroacetic acid was added (5% final conc.) followed by 15 min incubation on ice. Protein pellets were obtained by centrifugation (13 000 rpm, 30 min, 4°C) and washed two times with ice-cold acetone (13 000 rpm, 15 min, 4°C). The supernatants were carefully removed and the pellets were allowed to air dry. Pellets were resuspended in individual volumes of SDS loading buffer relative to the OD_600_ measurements of the respective culture.

### Sample collection for RNA-seq analyses

RNA-seq experiment to identify VqmR targets: Biological triplicates of Δ*vqmR* cells carrying either the pBAD-Ctr or the pBAD-*vqmR* plasmid were grown to OD_600_ = 0.5 in LB media. Cells were treated with 0.2% (final conc.) l-Arabinose and harvested after 15 min. Addition of Stop Mix (95% [vol/vol] EtOH and 5% [vol/vol] phenol) terminated ongoing transcription and translation. The samples were frozen in liquid nitrogen and stored at –80°C until RNA preparation. Autoinducer RNA-seq: Biological triplicates of a Δ*luxS, cqsA, tdh* triple mutant strain were grown overnight in M9 minimal media, supplemented with single or combinations of the following autoinducers (5 μM final conc. each): autoinducer 2 (AI-2), cholera-autoinducer-1 (CAI-1), or 3,5-dimethylpyrazin-2-ol (DPO) or water (mock). Bacteria were diluted 1:500 in fresh media containing the same autoinducers and samples were collected at OD_600_ = 0.2. Stop Mix (95% EtOH, 5% phenol, [vol/vol]) prevented further transcription and translation. Cells were pelleted (4000 rpm, 15 min, 4°C), resuspended in Tri-reagent (Sigma) and stored at –80°C until further processing.

### Construction of cDNA libraries and Illumina sequencing

Total RNA was digested with DNaseI and depletion of ribosomal RNA was performed using the Ribo-Zero kit (Epicentre) for Gram-negative bacteria. Integrity of the prepared RNA was confirmed using an Agilent 2100 Bioanalyzer. Directional cDNA libraries were prepared using the NEBNext Ultra II Directional RNA Library Prep Kit for Illumina (NEB E7760) according to the manufacturer's instructions and cDNA library quality was tested on an Agilent 2100 Bioanalyzer. The libraries were sequenced using a HiSeq 1500 machine (Illumina) in single-read mode with 100 bp read length. The sequencing data has been deposited at Gene Expression Omnibus (GEO) under the GSE115711 accession code.

## RESULTS

### Identification and validation of additional VqmR target mRNAs

QS regulates hundreds of genes in *Vibrios* (33) and global transcriptome profiles show that QS-controlled genes can be classified into low- and high-cell density. In our previous work, we used high-cell density cultures and VqmR pulse expression followed by global transcriptome analysis to identify target mRNAs of VqmR in *V. cholerae* (24). To identify additional targets of VqmR, here we investigated exponentially growing cells (OD_600_ = 0.5) and scored global transcriptome changes using RNA-sequencing. Differentially expressed genes were determined by comparing cells induced for VqmR expression from a pBAD promoter for 15 min to an empty vector control. These analyses identified 11 mRNAs showing at least 2.5-fold regulation by VqmR (Table 1). VqmR-mediated repression of five transcripts (*vpsT, vca0068, vc1865, vc1063* and *vca0591-vca0590*) was also observed in our previous analyses (24), supporting our approach. Newly identified target candidates included the mRNAs of two conserved hypothetical proteins (*vc0789* and *vc0865*), *ulaA* (encoding part of an ascorbate transport system), *ndk* (encoding nucleoside diphosphate kinase), as well as *aphA* (encoding a major transcriptional regulator of QS in *V. cholerae*, see Figure 1). All transcripts were repressed by VqmR, which was also confirmed using quantitative real-time PCR (Supplementary Figure S1A).

**Table 1.** Genes differentially regulated by VqmR pulse expression

Gene	Description^a^	Fold change^b^
*vc0789*	Hypothetical protein	−12.0
*vc1865* ^c^	Hypothetical protein	−9.6
*vca0068* ^c^	Methyl-accepting chemotaxis protein	−5.7
*vc0865*	Hypothetical protein	−3.4
*ulaA*	PTS system ascorbate-specific transporter	−3.3
*vca0591* ^c^	Peptide ABC transporter	−3.2
*vca0590* ^c^	Peptide ABC transporter permease	−3.0
*vc1063* ^c^	Acyl-CoA thioesterase II	−2.9
*aphA*	PadR family transcriptional regulator	−2.8
*ndk*	Nucleoside-diphosphate kinase	−2.5
*vpsT* ^c^	LuxR family transcriptional regulator	−2.5

^a^Description based on the annotation at KEGG (https://www.genome.jp/kegg).

^b^Fold change obtained by transcriptomic analysis of pBAD-driven VqmR expression using RNA-seq. Genes that were at least 2.5-fold differentially regulated and were statistically significant (Bonferroni ≤ 1E–10) are listed.

^c^VqmR target genes previously reported in (24).

**Figure 2.**
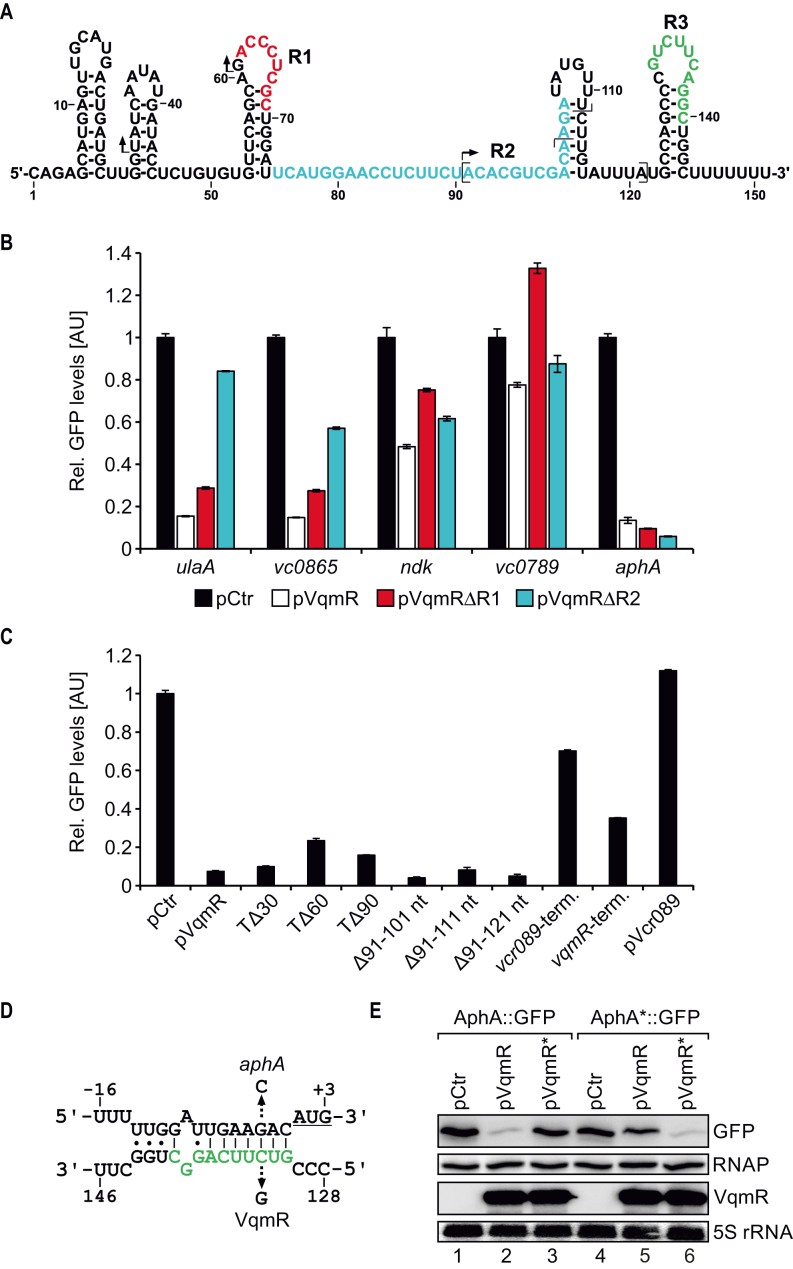
VqmR target genes and base-pairing of VqmR with the *aphA* 5′ UTR. (**A**) Secondary structure of VqmR (24). The VqmR base-pairing sequences are highlighted in red (R1), blue (R2) and green (R3). Arrows and brackets mark the truncation start sites and the internal deletion regions investigated in C, respectively. (**B**) *E. coli* harbouring plasmids carrying the five genes denoted on the x-axis each fused to *gfp* were co-transformed with a control plasmid (pCtr) or the indicated VqmR expressing plasmids. Transcription of *vqmR* and *gfp* was driven by constitutive promoters. Cells were cultivated in LB to OD_600_ = 0.5 and GFP production was measured. GFP levels of strains carrying the control plasmid were set to 1. Error bars represent the SD of three biological replicates. (**C**) *E. coli* cells carrying the *aphA::gfp* reporter were tested for repression by various VqmR mutants. Cells were grown in LB to OD_600_ = 0.5 and GFP production was measured. Error bars indicate the SD of three biological replicates. (**D**) Predicted base-pairing of the VqmR R3 sequence (green) with the 5′ UTR of *aphA*. The arrows indicate the single nucleotide mutations tested in E and the start codon is underlined. (**E**) Repression of AphA::GFP and AphA*::GFP (G-3C) by VqmR and VqmR* (C133G). Cells were grown in LB to OD_600_ = 0.5 and GFP levels were measured using Western Blot. RNAP served as the loading control.

Previous work focussing on the molecular mechanism of VqmR-mediated gene regulation showed that the VqmR sRNA employs one of two conserved domains (R1 and R2, see Figure 2A) to base-pair with target mRNAs (24). To test if the newly identified targets, i.e. *vc0789, vc0865, ulaA, ndk* and *aphA*, were also regulated at the post-transcriptional level by VqmR, we used a well-established GFP-based reporter system tailored to score post-transcriptional gene control in bacteria (39). In this system, the 5′ UTR (untranslated region) and the sequence corresponding to the first 20 amino-acids of the target genes are fused to *gfp* under the control of the P_TetO_ promoter. These plasmids were introduced into *Escherichia coli* along with a second plasmid expressing the *vqmR* gene from a P_Tac_ promoter. We discovered significantly reduced GFP production for all five candidate targets when VqmR was present (Figure 2B). We repeated these experiments in an *E. coli* strain lacking *hfq*, and no target regulation occurred (Supplementary Figure S1B). To investigate which of the two conserved base-pairing domains of VqmR mediated target repression, we individually deleted the R1 and R2 sequences in *vqmR* and measured GFP production. We discovered that repression of *vc0865* and *ulaA* was significantly impaired in the absence of domain R2, while down-regulation of *ndk* and *vc0789* was impaired when domain R1 was removed. Unexpectedly, VqmR-mediated repression of *aphA* did not require either of the two base-pairing domains (Figure 2B).

### VqmR interacts with *aphA* via a third base-pairing domain

The data presented in Figure 2B suggested that VqmR inhibits *aphA* by base-pairing using some unknown sequence element of VqmR. To test this possibility, we generated truncated VqmR variants, *i.e*. we deleted the first 30, 60 and 90 nucleotides of VqmR (these VqmR variants are called TΔ30, TΔ60, and TΔ90, respectively) and monitored AphA::GFP levels. None of these mutants abrogated VqmR repression (Figure 2C, bars 1–5). In addition, we also constructed internal deletions in *vqmR*, removing nucleotides 91–101, 91–111 and 91–121. Again, these mutants did not affect repression of AphA::GFP (Figure 2C, bars 6–8).

These results showed that none of the canonical base-pairing sequences of VqmR are involved in *aphA* repression and led us to conclude that VqmR-mediated repression of *aphA* possibly depended on a sequence element located in the Rho-independent terminator of VqmR. To probe this hypothesis, we exchanged the terminator of *vqmR* with the terminator sequence of an unrelated sRNA of *V. cholerae*, named Vcr089 (24). Although the level of production of this chimeric sRNA was comparable to wild-type VqmR (Supplementary Figure S2A), repression of AphA::GFP was significantly reduced (∼1.7-fold versus ∼13.5-fold; Figure 2C, bar 9 versus 2). In the reciprocal experiment, we exchanged the terminator of *vcr089* with the *vqmR* terminator and discovered that AphA::GFP repression increased to ∼3-fold (Figure 2C, bar 10), while Vcr089 itself had no effect on AphA::GFP levels (Figure 2C, bar 11). Of note, the limited repression of AphA::GFP by the Vcr089 sRNA carrying the VqmR terminator (bar 10) might well be explained by the reduced stability of this chimeric sRNA when compared to the native VqmR sRNA (Supplementary Figure S2A).

These experiments prompted us to search for a possible base-pairing interaction using the RNA hybrid algorithm (40) with the *aphA* 5′ UTR and the VqmR terminator sequence as inputs. Indeed, these analyses revealed a potential RNA duplex involving the loop of the VqmR terminator element and the sequence directly upstream of the *aphA* start codon (Figure 2D). To test this prediction, we altered cytosine to guanine at position 133 of VqmR and measured production of AphA::GFP (Figure 2E and Supplementary Figure S2B). Mutation at this position strongly reduced AphA::GFP repression, while sRNA levels were unaffected. Likewise, a compensatory mutation from guanine to cytosine at position –3 of *aphA*::*gfp* fully restored repression by the mutated VqmR, whereas repression by wild-type VqmR was inhibited (Figure 2E and Supplementary Figure S2B). Thus, VqmR uses its Rho-independent terminator, and specifically the loop sequence, to repress AphA production. In accordance with the previously defined base-pairing sequences of VqmR, we termed this sequence R3 (Figure 2A).

The data presented in Figure 2D and E suggested that VqmR inhibits *aphA* by sequestering its RBS, which will block translation initiation and consequently reduce protein levels. To test this hypothesis, we first mutated the Shine-Dalgarno element in the RBS of the *aphA::gfp* reporter at three consecutive positons and monitored AphA::GFP levels. In all three cases, GFP production was strongly reduced (Supplementary Figure S3A). Next, we performed toeprinting analysis (41) of the *aphA* mRNA (Supplementary Figure S3B). Addition of purified 30S ribosomes along with initiator tRNA^fMet^ to the *aphA* mRNA resulted in a termination signal located 16 nucleotides downstream of translation initiation, which is in accordance with the annotated AUG start codon. To mimic base-pairing of VqmR at the predicted position in *aphA*, we used an LNA (locked nucleic acid) oligonucleotide matching to the VqmR seed sequence of VqmR-*aphA* RNA duplex (corresponding to nucleotides 131–138 of VqmR; compare Figure 2D and Supplementary Figure S3A). Indeed, titration of the LNA oligonucleotide reduced 30S binding in a concentration dependent manner and led to the detection of a second termination signal corresponding to the VqmR binding site (Supplementary Figure S3B). Together, our *in vivo* and *in vitro* data indicate that interaction with the VqmR sRNA inhibits translation initiation of the *aphA* mRNA and that VqmR competes with 30S ribosomes for binding of the *aphA* RBS.

### VqmR and DPO inhibit AphA protein production

**Figure 3.**
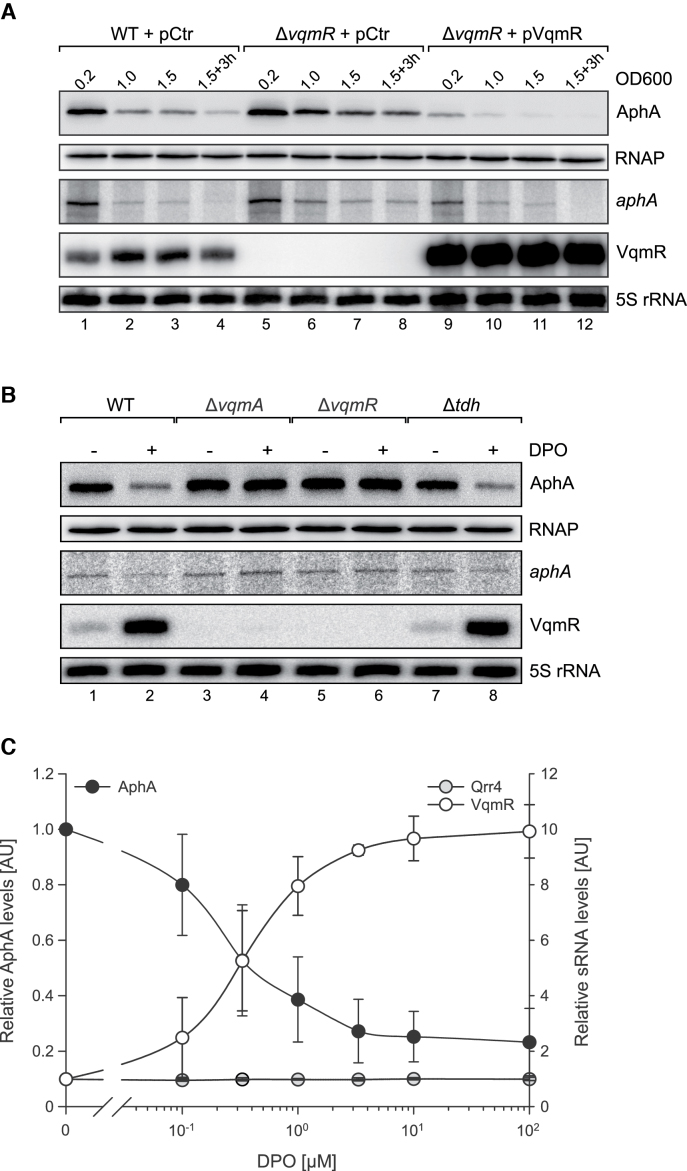
DPO inhibits AphA production. (**A**) *V. cholerae* wild-type and *vqmR* mutants carrying the indicated plasmids were cultivated in M9 minimal media supplemented with casamino acids (0.4% final conc.). At the indicated growth phases, total RNA and protein samples were collected. AphA::3XFLAG production was monitored on Western Blots and RNAP served as the loading control. VqmR and *aphA::3XFLAG* mRNA levels were probed on Northern Blots using 5S rRNA as loading control. (**B**) Total RNA and protein samples were collected from *V. cholerae* wild-type, Δ*vqmA*, Δ*vqmR* and Δ*tdh* strains at low cell density (OD_600_ = 0.2). Cells were cultivated in M9 minimal media and one set of cultures was supplemented with DPO (100 μM final conc.). AphA::3XFLAG production was determined using Western Blot. Northern Blot was used to probe the expression of *aphA-3XFLAG* and VqmR. RNAP and 5S rRNA served as loading controls for the Western and Northern Blot analyses, respectively. (**C**) *V. cholerae* Δ*tdh* cells were cultivated in M9 medium supplemented with the indicated DPO concentrations (x-axis) and total RNA and protein samples were harvested at low cell densities (OD_600_ = 0.2). AphA production was analyzed on Western Blots (left y-axis), sRNA levels (VqmR and Qrr4) were determined on Northern Blots (right y-axis). Error bars represent the SD of five (AphA) and three (sRNAs) biological replicates, respectively.

Next, we were interested to test the effect of VqmR on AphA protein production *in vivo*. To this end, we engineered an *aphA::3XFLAG* construct and introduced it onto the chromosome of *V. cholerae* wild-type and Δ*vqmR* strains at the *aphA* locus. These strains were transformed with either a vector control (pCtr) or a VqmR over-expression plasmid (pVqmR) and cultivated in M9 minimal medium containing casein acid hydrolysate (casamino acids) as a threonine source for DPO production. At selected time-points, total RNA and protein samples were collected and examined by Northern and Western blotting, respectively. As expected, in wild-type, levels of AphA protein decreased at high cell density along with a reduction in *aphA* mRNA abundance (Figure 3A, lanes 1–4). In *V. cholerae* cells lacking *vqmR*, AphA protein levels remained unaffected at low cell density (OD_600_ of 0.2), which was also recapitulated at the *aphA* mRNA level and is in accordance with limited VqmR expression under this condition (Figure 3A, lane 1 vs. 5). At higher cell densities (OD_600_ of 1.0, 1.5 and 3 h after cells reached an OD_600_ of 1.5), AphA protein levels were ∼3–4-fold higher in the *vqmR* mutant when compared to wild-type cells (lanes 2–4 versus 6–8). In contrast, *V. cholerae* Δ*vqmR* cells over-expressing VqmR displayed significantly reduced AphA protein and mRNA levels under all conditions (Figure 3A, lane 9–12). Of note, plasmid-borne VqmR expression had a stronger effect on AphA protein levels, when compared to the reduction in *aphA* mRNA. These results could indicate that, when over-expressed, VqmR-mediated repression of *aphA* acts predominantly by inhibiting translation initiation.

Elevated levels of AphA in Δ*vqmR* cells cultivated to high cell density (Figure 3A) indicated that VqmR has a negative effect on AphA levels when DPO accumulates in the environment. To explore this possibility, we cultivated *V. cholerae* cells in M9 minimal medium lacking amino-acids (eliminating endogenous DPO production) to low cell density (OD_600_ = 0.2) and compared AphA production in the presence and absence of exogenously supplied synthetic DPO (100 μM final conc.). In wild-type cells, addition of DPO reduced AphA levels by ∼3-fold, which was also corroborated at the *aphA* mRNA level (Figure 3B, lane 1 versus 2). As expected, AphA protein and its mRNA did not change in response to DPO in *V. cholerae* cells lacking either *vqmA* or *vqmR* (Figure 3B, lanes 3–6). However, DPO-mediated repression of AphA occurred in cells lacking *tdh* (Figure 3B, lanes 7–8), which is required for DPO synthesis but not for DPO detection or signal transduction (Figure 1). In line with these observations, exogenously added DPO activated VqmR production in wild-type and Δ*tdh* cells, while no VqmR was detected in the *vqmA* and *vqmR* mutants. These data show that DPO inhibits AphA production in *V. cholerae* and that the DPO-receptor, VqmA and the VqmR sRNA mediate this phenotype.

We previously showed that DPO accumulates in cell-free supernatants of *V. cholerae* at a concentration of ∼1 μM (22). To test if endogenous levels of DPO would also inhibit AphA production, we performed a titration experiment in which we gradually increased the levels of synthetic DPO and tested AphA and VqmR levels on Western and Northern Blots, respectively (Figure 3C). We discovered that DPO concentrations as low as 0.33μM significantly increased VqmR production, which also resulted in a ∼2-fold reduction in AphA production. At a concentration of 1 μM DPO, repression of AphA increased to ∼2.5-fold and reached a maximum of ∼3-fold when higher concentrations of DPO were used. Importantly, this saturation in AphA repression coincided with the maximal VqmR expression, while DPO titration did not affect Qrr4 production (Figure 3C).

### DPO down-regulates virulence gene expression in *V. cholerae*

**Figure 4.**
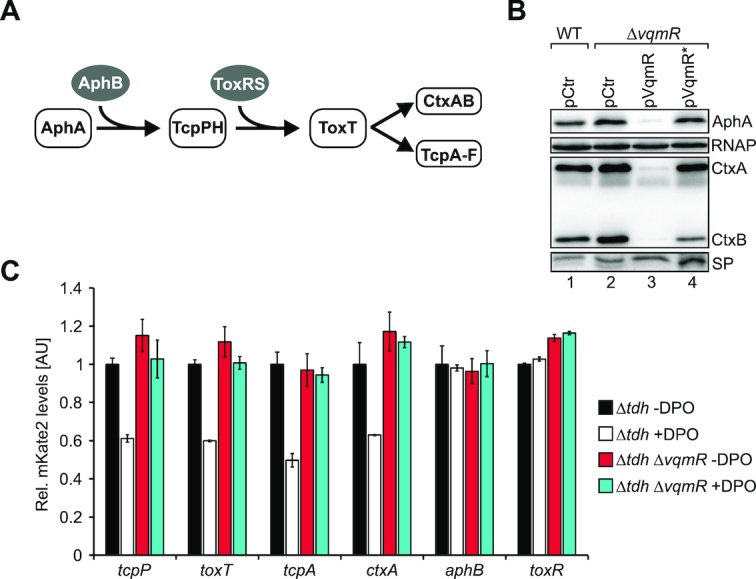
DPO and VqmR inhibit virulence gene expression. (**A**) The virulence cascade of *V. cholerae*. (**B**) *V. cholerae* wild-type and Δ*vqmR* strains carrying the indicated plasmids were cultivated under AKI conditions. Cellular and secreted protein (SP) fractions were harvested 2h and 16 h after switching from static to aerating conditions, respectively and tested for AphA-3XFLAG and CtxAB production on Western Blots. RNAP and a coomassie-stained SDS gel (bottom) confirmed equal loading of the two protein fractions. (**C**) *V. cholerae* Δ*tdh* or Δ*tdh, vqmR* cells carrying plasmids with the indicated transcriptional reporters were cultivated under AKI conditions in the presence or absence of DPO (100 μM final conc.) and fluorescence was measured 2 h after switching to aerating conditions. mKate2 levels of the Δ*tdh* cells cultivated without DPO were set to 1. Error bars represent the SD of three biological replicates.

AphA is a key regulator of virulence gene expression in *V. cholerae* and required for intestinal colonization in an infant mouse model of infection (30). In concert with AphB, AphA induces the expression of the transmembrane regulators TcpP and TcpH (42). TcpPH and another transmembrane regulator, ToxRS, activate the production of ToxT, which finally induces the expression of *ctxAB* and *tcpA-F* (Figure 4A and (43)).

To investigate the role of DPO-mediated gene control in virulence gene expression in *V. cholerae*, we first tested the effect of VqmR on CtxAB and AphA protein production. Specifically, we cultivated *V. cholerae* wild-type and Δ*vqmR* cells, both carrying a vector control, in AKI medium to induce virulence factor production (36). We discovered a modest (∼1.5-fold) increase in AphA levels in the absence of *vqmR* (Figure 4B, lane 1 versus 2). In contrast, a *vqmR* mutant strain carrying *vqmR* on a multi-copy plasmid strongly reduced AphA levels (∼13-fold), when compared to wild-type *V. cholerae* (Figure 4B, lane 1 versus 3). Likewise, VqmR over-production down-regulated CtxA and CtxB levels by ∼18.2-fold and ∼27.6-fold, respectively. To obtain additional evidence that base-pairing of VqmR with the *aphA* mRNA caused AphA and CtxAB repression, we also tested the effect of the VqmR point-mutant showing strongly reduced AphA::GFP repression (Figure 2D and E) on AphA and CtxAB production. We discovered that the mutated VqmR variant failed to inhibit AphA and CtxAB production (Figure 4B, lane 4). Together, these data suggest that VqmR-mediated repression of *aphA* prevents virulence gene expression in *V. cholerae*.

To monitor the activity of *V. cholerae* virulence genes in the context of DPO, we generated mKate2-based transcriptional reporters to all genes of the cascade (Figure 4A) and transformed these constructs into *V. cholerae* cells lacking *tdh* (to eliminate endogenous DPO production) and cells lacking *tdh* and *vqmR*. Again, we used AKI medium to induce virulence gene expression, however, for these experiments, one set of cultures was supplemented with synthetic DPO (100 μM final conc.). Our data showed that DPO significantly inhibited the promoter activities of *tcpP, toxT, tcpA*, and *ctxA*, however, as expected, did not affect the promoters of *aphB* and *toxR* (Figure 4C). These results were specific to DPO-mediated activation of *vqmR*, since DPO failed to down-regulate the promoters of *tcpP, toxT, tcpA*, and *ctxA* in the Δ*tdh vqmR* double mutant (Figure 4C). Therefore, we conclude that the DPO-controlled QS pathway negatively affects the production of virulence factors in *V. cholerae*.

### The three autoinducers of *V. cholerae* act together to control AphA production

**Figure 5.**
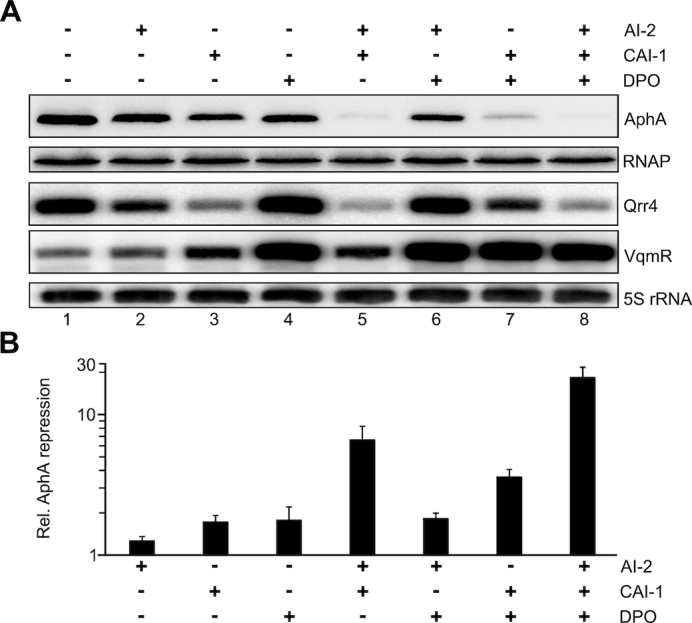
AI-2, CAI-1 and DPO act in concert to repress AphA production. (**A**) The *V. cholerae* Δ*luxS, cqsA, tdh* mutant was cultivated in M9 minimal media containing the indicated autoinducers (5 μM final conc. each) to OD_600_ = 0.2. AphA::3XFLAG, Qrr4 and VqmR levels were monitored on Western Blots and Northern Blots, respectively. RNAP (Western Blot) and 5S rRNA (Northern Blot) served as loading controls. (**B**) Quantification of (A). AphA levels in the mock-treated sample was set to 1. Error bars represent the SD of three biological triplicates.

There are currently three autoinducers known in *V. cholerae* (11,12,22). Whereas AI-2 and CAI-1 act through LuxO and the Qrr sRNAs to activate *aphA*, DPO functions through VqmA and VqmR to repress *aphA* (Figure 1B). Importantly, although VqmR and the Qrr sRNAs act antagonistically on *aphA*, all three autoinducers inhibit *aphA* production since AI-2 and CAI-1 inhibit production of the Qrr sRNAs which are activators of *aphA*, and DPO activates VqmR transcription, which directly represses *aphA*. To test the individual contributions of the autoinducers on AphA production, we cultivated a *luxS, cqsA, tdh* triple mutant in M9 minimal medium and added AI-2, CAI-1 and DPO at saturating concentrations (5 μM final conc.). We collected total protein and RNA samples at low cell density (OD_600_ = 0.2) and probed AphA protein levels on Western Blots, as well as Qrr4 and VqmR production on Northern Blots (Figure 5A). We discovered that AI-2, although reducing Qrr4 levels by ∼2.2-fold (Figure 5A, lane 1 versus 2), did not significantly reduce AphA levels (Figure 5A and B). CAI-1 and DPO both inhibited AphA by ∼1.8-fold (Figure 5B). CAI-1 reduced Qrr4 levels by ∼5.6-fold, whereas DPO did not affect Qrr4 (Figure 5A, lanes 1, 3, 4). As expected, VqmR expression was strongly induced by DPO (∼15-fold), but remained unaffected by AI-2 (Figure 5A, lanes 1, 2, 4). Interestingly, CAI-1 also slightly activated (∼2.8-fold) VqmR production (lane 1 versus 3). We currently do not understand the molecular mechanism underlying CAI-1-mediated VqmR induction and whether this regulation is biologically relevant.

The genetic setup of *V. cholerae*’s QS circuit (Figure 1) suggested that the three autoinducers act together to control AphA production. To test this hypothesis, we monitored the collective effect of the autoinducers on AphA, Qrr4 and VqmR levels. Combination of AI-2 and CAI-1 inhibited AphA by ∼6.6-fold and reduced Qrr4 levels by ∼8.8-fold when compared to the untreated sample (Figure 5A and B). In contrast, AphA and Qrr4 levels of cells treated with AI-2 and DPO were similar to those treated with DPO only, suggesting that CAI-1 has a stronger effect on Qrr4 production, when compared to AI-2 (16). In line with this observation, the combination of CAI-1 and DPO resulted in a more robust (∼3.6-fold) reduction in AphA, when compared to AI-2 and DPO (Figure 5A, lanes 1, 6, 7). Finally, we also tested the combined effect of all three autoinducers. Indeed, addition of AI-2, CAI-1 and DPO boosted AphA repression to 18.3-fold along with the expected reduction in Qrr4 levels (∼10-fold) and activation of VqmR (∼11-fold).

To corroborate these results, we also performed the reciprocal experiments, i.e. we generated single, double and triple deletion strains of the autoinducer synthase genes (*luxS, cqsA* and *tdh*) and monitored AphA production in late stationary phase cells (6 h after cells reached an OD_600_ of 1.5) using Western Blot analysis (Supplementary Figure S4). When compared to wild-type *V. cholerae*, all three single mutants displayed a significant increase in AphA levels with Δ*cqsA* showing the strongest up-regulation (∼7.3-fold, Supplementary Figure S4, lane 1 versus 3). Mutation of *luxS* or *tdh* both increased AphA production by ∼3-fold (Supplementary Figure S4, lanes 2 and 4). *V. cholerae* strains lacking two of the synthases, i.e. Δ*luxS*, *cqsA*; Δ*luxS*, *tdh* and Δ*cqsA*, *tdh*, all showed elevated production of AphA, when compared to the relevant mutants lacking only one of the synthase genes (Supplementary Figure S4, lanes 5–7). Finally, deletion of all three autoinducer synthase genes boosted AphA levels by >15-fold (Supplementary Figure S4, lane 8), which was the strongest effect detected in our panel.

Together our data show that QS-mediated down-regulation of AphA in *V. cholerae* is enhanced by the combined action of multiple autoinducers acting through LuxO and VqmA to modulate production of the Qrr and VqmR sRNAs.

### Global transcriptome analysis of autoinducer function in *V. cholerae*

The combined regulatory effect of AI-2, CAI-1 and DPO on AphA levels prompted us to probe autoinducer functions in *V. cholerae* at a larger scale. Specifically, we used the setup of the previous experiments (Figure 5A) and RNA-sequencing to monitor autoinducer-controlled changes at a transcriptome-wide level. Again, we performed these experiments in Δ*luxS, cqsA, tdh* cells and added single or combinations of the autoinducers at saturating levels (5 μM final conc.) to identify the full set of autoinducer-responsive genes in *V. cholerae*.

**Figure 6.**
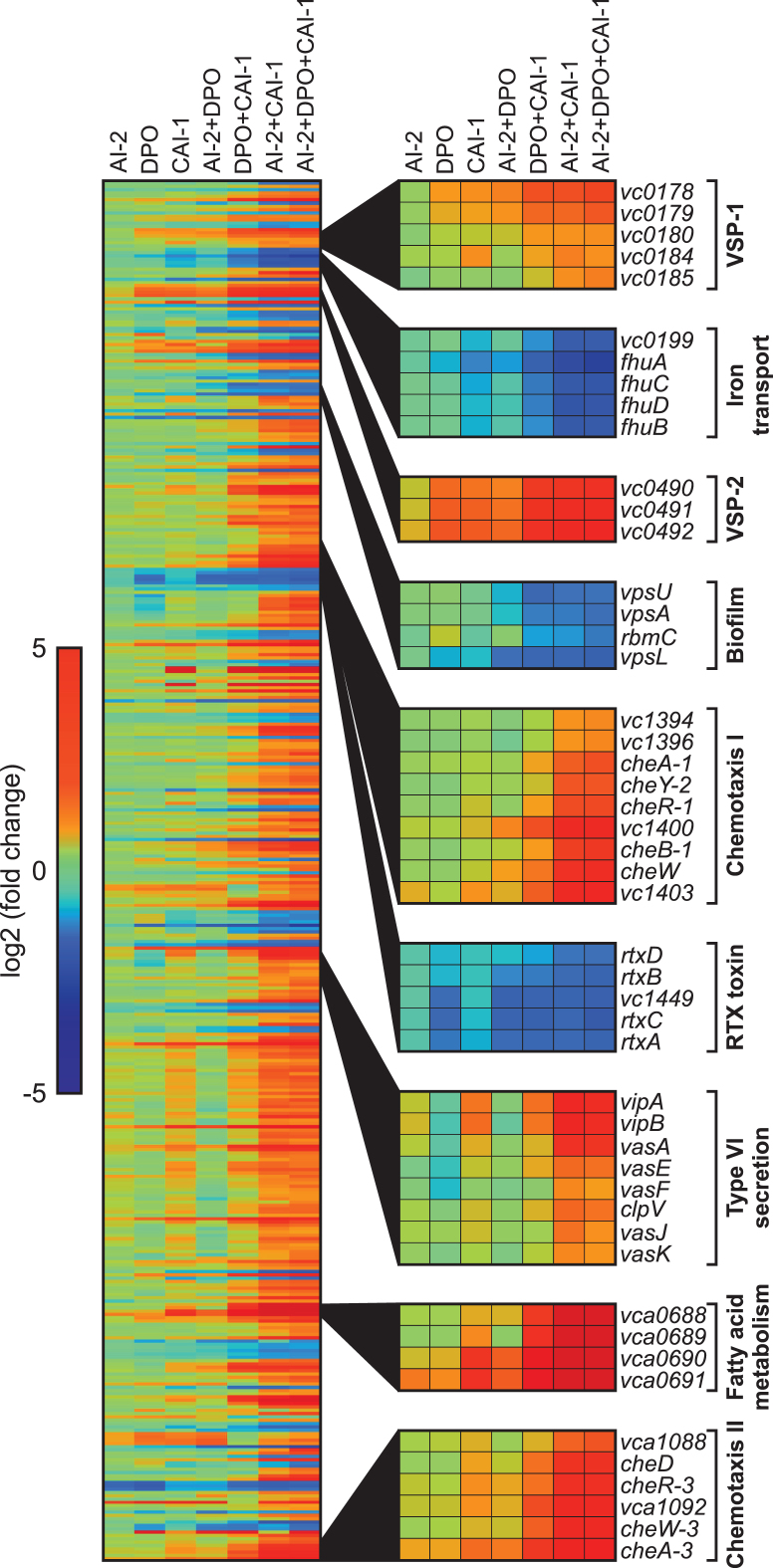
Genome-wide transcriptome changes in response to the AI-2, DPO and CAI-1 autoinducers. Heatmap displaying 420 genes differentially expressed (≥2-fold) in response to at least one of the autoinducers. *V. cholerae* Δ*luxS, cqsA, tdh* cells were cultivated in M9 minimal media containing single or combinations of the autoinducers (5 μM final conc. each). Selected gene clusters showing significant regulation are highlighted on the right. Fold changes of the normalized expression values were calculated relative to the normalized expression values of the mock treated replicates.

In line with our above results (Figure 5B), autoinducer-mediated repression of *aphA* was most prominent when all three autoinducers were supplemented (∼11-fold), whereas addition of single autoinducers resulted in modest repression (Supplementary Table S1). In general, transcriptome analysis revealed only very few (4) differentially expressed genes (≥2-fold) in response to AI-2 and moderate changes (40 and 60 genes) when cells were exposed to DPO or CAI-1, respectively ( Figure 6 and Supplementary Table S1). Treatment of *V. cholerae* with two autoinducers significantly enhanced QS-mediated gene regulation. Combination of AI-2 and CAI-1 had the strongest effect leading to the differential expression of 323 genes, followed by DPO/CAI-1 (151 genes), and AI-2/DPO (59 genes). Together, AI-2, DPO and CAI-1 rendered almost 400 genes (Supplementary Table S1), suggesting that all three autoinducers are required for a full QS response. For example, genes from two genetic islands specific to *V. cholerae* strains of the 7th pandemic (VSP-1 and VSP-2) were not affected by AI-2, mildly activated by DPO and CAI-1, and strongly induced by combined treatment with the three autoinducers (Figure 6). We discovered similar expression patterns for genes associated with fatty acid metabolism (*vca0688*-*vca0691*), as well as genes located in two chemotaxis clusters of *V. cholerae* (*vc1394*-*vc1403* and *vca1088*-*cheA-3*). QS-mediated activation of chemotaxis genes in *V. cholerae* is in accordance with a previous report (44).

We also discovered reciprocal expression patterns, i.e. downregulation in response to the autoinducers. For example, genes relevant for iron transport (*vc0199*-*fhuB*), biofilm formation (*vpsU, vpsA, rbmC* and *vpsL*) and RTX toxin secretion (*rtxA-D*) were all repressed in response to the autoinducers. Of note, DPO alone also significantly repressed the *rtx* operon, which is in accordance with our previous work showing VqmR-mediated repression of *rtx* (24). Interestingly, DPO by itself seems to function as a repressor of type VI secretion genes, however, this regulation can be overcome by CAI-1 but not by AI-2 (Figure 6).

## DISCUSSION

Gene regulation by QS is crucial for virulence factor production and collective functions of various bacterial pathogens, including *V. cholerae* (26). For many bacterial pathogens, QS relies on multiple signalling molecules, however, how these act together to control gene expression is frequently unknown (2). Given that enteric pathogens such as *V. cholerae* regularly interact with other species, e.g. during the course of an infection, one can predict that exposure to multiple signalling molecules is the norm rather than the exception. Therefore, studying the influence of autoinducer mixtures on gene expression and behaviour of bacterial pathogens is fundamental to develop a global understanding of QS functions in these organisms.

In this study, we discovered that the DPO autoinducer inhibits the production of AphA, a central regulator of virulence gene expression in *V. cholerae*. Repression of AphA by DPO requires the VqmA receptor protein, as well as the VqmR sRNA (Figure 3B). VqmR belongs to the large group of Hfq-dependent sRNAs (23). These sRNAs control gene expression by base-pairing with target mRNAs, which can either repress or activate gene expression (45,46). Repression of *aphA* by VqmR relies on a base-pairing sequence that we discover here to be located in the loop of the Rho-independent terminator stem of the sRNA (Figure 2A). There are only few documented cases of target recognition via the terminator sequence of an Hfq-dependent sRNA. For example, base-pairing of the OxyS sRNA with the *fhlA* mRNA requires two independent sequence elements, one of which is located in the loop of the terminal stem in the sRNA (47). Rho-independent terminator sequences have been shown to recruit Hfq to sRNAs (48) and are considered important for transcript stability providing protection from 3′-5′ exonucleolytic degradation (49). We speculate that base-pairing with *aphA* could destabilize the terminator structure of VqmR and thereby facilitate turn-over of the sRNA. Indeed, previous work focussing on the regulatory mechanisms of the Qrr sRNAs in *Vibrio harveyi* revealed that base-pairing with specific target mRNAs affects Qrr degradation and thereby modulates QS fidelity (50). One of the target mRNAs relevant for Qrr turn-over is *aphA*, which is also regulated by VqmR. However, the Qrr sRNAs and VqmR have antagonizing effects on *aphA*, with the Qrr sRNAs acting to increase AphA production (19,51), whereas VqmR reduces AphA levels (Figure 3A). It will be interesting to test how the two sRNAs compete for *aphA* regulation and if the molecular mechanisms underlying post-transcriptional control of this mRNA will provide priority to regulation by one of the sRNAs. Sequence alignment of the *aphA* 5′ UTR showed that the base-pairing sites of VqmR and the Qrr sRNAs are conserved among *Vibrios* (Supplementary Figure S5) and so are the relevant interaction sites in Qrr2-4 (51), as well as VqmR (Figure 2A). Therefore, regulation of *aphA* by two competing sRNAs species could be relevant for collective functions of many *Vibrio* strains.

Regulation of *aphA* by VqmR links the AI-2/CAI-1 and DPO QS pathways at a critical point as AphA controls virulence by activating *tcpPH* (42), modulates QS by repressing *hapR* (19), and enhances biofilm formation by inducing *vpsT* transcription (32). Activation of *vpsT* transcription by AphA, together with post-transcriptional repression of *aphA* (Figure 2B) and *vpsT* (24) by VqmR indicates the presence of a type 2 coherent feed-forward loop (52) controlling VpsT production in *V. cholerae* (Figure 1B). In this scenario, VqmR acts on top of the cascade repressing *vpsT* translation by base-pairing to the mRNA, while repressing *vpsT* transcription by reducing AphA levels. An increasing number of regulatory RNAs are being recognized to participate in mixed network motifs with transcription factors (53). For example, the Spot42 sRNA of *E. coli* together with the CRP transcriptional regulator forms a multi-output feed-forward loop to decrease leaky expression of target genes (54). In *Salmonella*, the RprA sRNA activates the expression of RpoS and RicI to prevent plasmid conjugation when the cell membrane is damaged (55). Coherent feed-forward loops typically reduce noise in biological systems (53) and in the case of VqmR-mediated repression of *aphA* and *vpsT* might help to facilitate transition between QS states and to coordinate virulence gene expression and biofilm formation in *V. cholerae*. Of note, VpsT activity is also controlled post-translationally by binding of c-di-GMP (56), which could add an additional layer of regulation.

How and when *V. cholerae* changes from one QS state to another depends on the accumulation of autoinducers in the environment (57). AI-2 and CAI-1 are recognized by the membrane-bound receptors, LuxPQ and CqsS, respectively and channel information into a shared signalling cascade (Figure 1). Importantly, both receptors function as kinases to phosphorylate LuxU in the absence of AI-2 and CAI-1, but convert to phosphatases when the autoinducers are bound. This logic prevents premature modulation of QS-responsive pathways when only a single autoinducer is present (21). Indeed, global gene expression analysis showed that, although provided at saturating concentrations (16), AI-2 and CAI-1 had only modest effects on the transcriptome of *V. cholerae*, when compared to a combination of the two autoinducers (Figure 6 and Supplementary Table S1). Our data are also in line with a recent report showing that CqsS exhibits a stronger phosphatase activity than LuxQ and supports the existence of a positive feedback loop upregulating *cqsS* levels by the autoinducers (16). Activation of *cqsS* was most prominent (∼2.5-fold) when all three autoinducers were present indicating a combined effect on *cqsS* expression (Supplementary Figure S6A).

Similarly, we also discovered autoinducer-mediated activation of the mRNAs encoding the VqmA and CqsR receptors (21), while the mRNAs of *luxPQ* and *vpsS* remained constant under all tested conditions (Supplementary Figure S6A). Activation of the VqmA production might also explain induction of VqmR expression in CAI-1 treated cells (Figure 5A). However, how CAI-1 influences *vqmA* expression is currently unclear. Transcripts encoding proteins involved in transduction of QS signals (*luxOU* and *vspV*) displayed only minor changes in response to the autoinducers (Supplementary Figure S6B), whereas mRNAs of downstream transcriptional regulators, i.e. *aphA, hapR* and *vpsT*, showed the expected expression patterns in response to the autoinducers (Supplementary Figure S6C). Modest upregulation (∼1.5-fold) of *luxOU* in cells treated with a combination of autoinducers supports previous reports suggesting that Qrr-mediated repression of *luxO* does not involve significant transcript turn-over (50,58). Strong activation of *hapR* by AI-2 and CAI-1 is expected due to Qrr-mediated repression of *hapR* (18), however, *hapR* levels were also activated by DPO (∼2-fold, Supplementary Figure S6C), which might be explained by negative regulation of the *hapR* promoter by AphA (19). Therefore, although only the Qrr sRNAs base-pair with *hapR* to inhibit translation, VqmR can promote similar regulation by repressing *aphA*. Indeed, adding CAI-1 and DPO to *V. cholerae* significantly increased *hapR* abundance when compared to cells treated with CAI-1 only (Supplementary Figure S6C). Together, these data support the hypothesis that the three autoinducer act in concert to modulate QS functions. Of note, naturally occurring frameshifts in *hapR* have been reported for several toxigenic *V. cholerae* isolates (59). Mutants lacking HapR are likely to lose most of their QS functions, however, regulation of *aphA* and *vpsT* by AI-2/CAI-1 and DPO possibly retains basic QS-mediated gene regulation in these strains.

Besides looking at known QS-mediated responses in *V. cholerae*, our transcriptomic approach also allowed us to investigate the QS response of additional sets of genes relevant for pathogenicity and collective behavior. Mapping of the transcriptome data to the *V. cholerae* genome revealed activation of genes located in two chemotaxis clusters (Figure 6 and (44,60)). We also discovered autoinducer-mediated regulation of several methyl-accepting chemotaxis proteins (MCPs), also known as chemoreceptors. The chromosomes of *V. cholerae* El Tor contain 45 potential MCPs (61), 22 of which were differently regulated by the autoinducers (Supplementary Figure S7A). Whereas 18 MCP genes were upregulated in response to the autoinducers, four genes were repressed. Among these, *vca0068* was previously reported to be expressed during the infection process of *V. cholerae* (62,63) and we have shown that *vca0068* is repressed by base-pairing with VqmR (24). The ligand of VCA0068 is currently unknown, but regulation of this gene by both the autoinducers might indicate a role for this MCP in QS transition.

Another key factor for virulence, biofilm formation, and overall physiology of *V. cholerae* is c-di-GMP (64). Our transcriptomic data revealed differential expression of genes associated with the production, degradation and binding of c-di-GMP, with the majority (19/23) being induced when the autoinducers were supplemented (Supplementary Figure S7B). There was no clear separation between diguanylate cyclases (DGCs) and phosphodiesterases (PDEs) showing upregulation or downregulation by the autoinducers. However, we did find significant overlap with previous work studying QS-mediated regulation of DGCs and PDEs in *V. cholerae*. For example, expression of *vc1086, vc1370, vca0080, vca0848* and *vc0965* was induced by the autoinducers, which is in accordance with elevated expression of these genes in *luxO* deficient *V. cholerae*, which fail to produce the Qrr sRNAs (3). By the same token, levels of *cdgA* (*vca0074*), encoding a DGC involved in biofilm formation of *V. cholerae* (65,66), were inhibited in cells treated with multiple autoinducers (Supplementary Figure S7B) and were similarly reduced in the *luxO* mutant (3). How exactly QS signals and cellular c-di-GMP levels are coordinated to control biofilm formation and other collective functions in *V. cholerae* is currently unknown. However, activation of the *aphA* promoter by c-di-GMP-bound VpsR (67), together with QS-mediated post-transcriptional control of *aphA* mRNA (Figure 1), support the idea that the inter- and intracellular signalling pathways are intimately connected in *V. cholerae* (68).

AphA is a crucial factor for pathogenicity of *V. cholerae*, and so is biofilm formation (26). Our discovery that DPO inhibits both virulence expression (Figure 4C) and biofilm formation (24) via VqmR-mediated repression of *aphA* and *vpsT*, respectively, predicts that DPO could be used to restrict *V. cholerae* infections. This hypothesis is fuelled by a previous report showing that *vqmA* mutants outcompete *V. cholerae* wild-type cells in colonization assays using germ-free mice and the commensal gut bacterium *Ruminococcus obeum* (69). *V. cholerae* cells lacking *vqmA* fail to produce VqmR and display increased VpsT (24) and AphA (Figure 3A) levels, which could reinforce biofilm formation and virulence gene expression. There is significant interest in development of QS manipulation strategies to promote and to terminate beneficial and harmful bacterial behaviours, respectively (2,70). DPO could serve as a scaffold for such therapies since, conceivably, simple DPO-precursors such as l-threonine could be used to enhance DPO production and consequently inhibit biofilm formation and virulence genes expression of *V. cholerae* in the small intestine.

## DATA AVAILABILITY

The sequencing data has been deposited at Gene Expression Omnibus (GEO) under the GSE115711 accession code.

## Supplementary Material

Supplementary Data
