# Supplementary material for: Three autoinducer molecules act in concert to control virulence gene expression in Vibrio cholerae
Source: Nucleic Acids Res. 2019 Jan 15;47(6):3171–83. doi: 10.1093/nar/gky1320 (PMC6451090; doi:10.1093/nar/gky1320)
Supplement: Supplementary Data [file gky1320_supplemental_files.zip › MS DPO AphA 2018 revised supplement v05.pdf]

Three autoinducer molecules act in concert to control virulence gene expression in *Vibrio cholerae*

Roman Herzog[1], Nikolai Peschek[1,2], Kathrin S. Fröhlich[1], Kilian Schumacher[1] and Kai Papenfort[1,2]

[1] Faculty of Biology I, Department of Microbiology, Ludwig-Maximilians-University of Munich, 82152 Martinsried, Germany

[2] Munich Center for Integrated Protein Science (CIPSM)

This supplement contains:

Supplementary Figure Legends

Supplementary Materials and Methods

Supplemental References

Figures S1-7

Tables S1-4

TABLE OF CONTENTS

Figure S1 Additional VqmR mRNA targets

Figure S2 Northern blot analysis of VqmR variants and VqmR-*aphA* base-pairing validation

FigureS3 VqmR inhibits 30S ribosome binding on the *aphA* mRNA

Figure S4 Quantitative analysis of AphA production in *V. cholerae* autoinducer synthase mutants

Figure S5 Sequence alignment of the *aphA* 5' UTR

Figure S6 Expression profiles of selected QS genes after autoinducer treatment

Figure S7 MCPs and c-di-GMP related genes that are differentally expressed in response to AI-2, CAI-1 and DPO

Supplementary Materials and Methods

Table S1 Global impact of the autoinducers AI-2, CAI-1, and DPO on the transcriptome of *V. cholerae*

Table S2 Bacterial strains used in this study

Table S3 Plasmids used in this study

Table S4 DNA oligonucleotides used in this study

Supplementary Figure Legends

Figure S1: Additional VqmR mRNA targets. (A) Transcript levels of VqmR targets were monitored using qRT-PCR. The Δ*vqmR* mutant strain harboured either a control plasmid (pCtr) or a plasmid carrying the *vqmR* gene under control of an inducible P_{BAD} promoter (pVqmR). Cells were cultivated in LB to OD_{600}=0.5. They were treated with 0.2 % (final concentration) L-arabinose for 15 min and collected afterwards. Relative transcript expression levels in the strain carrying the control plasmid were set to 1. Error bars indicate the SD of three replicates. (B) VqmR regulation of target mRNAs was tested in *E. coli* Δ*hfq* cells. A control plasmid (pCtr) or a *vqmR* overexpression plasmid (pVqmR) was cotransformed with a post-transcriptional reporter plasmid that harboured an in frame fusion of the 5' UTR of the indicated target gene to *gfp*. Expression of the sRNA and the reporter fusions were driven by two constitutive promoters. Fluorescence was measured at OD_{600}=0.5 and GFP levels of the strain carrying pCtr were set to 1. Error bars represent the SD of three biological replicates.

Figure S2: Northern blot analysis of VqmR variants and VqmR-*aphA* base-pairing validation. (A) Upper part: Schematic drawings of the VqmR variants tested in Fig. 2C. VqmR sequences are illustrated as black lines, Vcr089 sequences are shown in blue lines. Truncated or internally deleted regions are depicted in grey. The numbers indicate the sizes of fragments truncated from the VqmR 5' end (TΔ30, TΔ60, TΔ90) or the nucleotide positions of internal nucleotide deletions (Δ91-101, Δ91-111, Δ91-121). The pink, green, and orange lines show the binding sites of the KPO-452, KPO-632, and the KPO-3688 oligonucleotide probes. Bottom part: Northern blots of the VqmR variants hybridized with a *vqmR*-terminator probe (green, KPO-632), a *vcr089*-terminator probe (orange, KPO-3688), and a probe binding the *vqmR*-backbone (pink, KPO-452). The triangles indicate the bands of the expected sizes that can be estimated by a DNA marker (M) provided on the left. 5S rRNA served as loading control. (B) Fluorescence measurements corresponding to the Western Blot shown in Fig. 2E. GFP levels of the strains carrying the empty control plasmid were set to 1. Error bars represent the SD of three biological replicates.

FigureS3: VqmR inhibits 30S ribosome binding on the *aphA* mRNA. (A) Schematic representation of the *aphA* mRNA segment containing the RBS and the AUG start codon (underlined). The interaction of the VqmR LNA and the *aphA* mRNA, which was investigated in B, is boxed in grey. Single nucleotide exchanges (indicated by arrows) were introduced in the RBS of the *aphA::GFP* reporter and tested for GFP produciton. GFP fluorescence was measured in exponentially growing cells (OD_{600}=0.5) and the fluorescence signal of the strain carrying the native RBS was set to 1. Error bars represent the SD of three biological replicates.

(B) 30S toeprint formation on 20 nM *aphA* mRNA was analysed by reverse transcription using a 5' end labelled, *aphA*-specific oligo (KPO-4077). Reactions contained 30S ribosomal subunits (20 nM), uncharged initiator tRNA-fMet (100 nM), and an LNA oligo mimicking the VqmR interaction (20, 200 and 1000 nM) where indicated. The position of the AUG start codon was mapped using a co-migrating sequencing ladder (G, A, T, and C lanes) generated with the same primer as used in the toeprinting reaction. The 30S/*aphA* toeprint signal and the premature termination signal at the LNA binding site are marked.

Figure S4: Quantitative analysis of AphA production in *V. cholerae* autoinducer synthase mutants. *V. cholerae* was harvested 6h after cells reached an OD_{600} of 1.5 in M9 minimal medium containing casein acid hydrolysate. AphA production in the indicated *V. cholerae* mutant cells was monitored using Western Blots, quantified, and compared to wild-type AphA levels. RNAP was used as a loading control.

Figure S5: Sequence alignment of *aphA* 5' UTR. The *aphA* 5' UTR sequences of several *Vibrio* species were aligned using the Multalin algorithm (1). Numbers above the sequences indicate the distance to the first nucleotide of the *aphA* start codon (denoted +1 and underlined, respectively). sRNA binding sites are boxed. Vch, *Vibrio cholerae* (NCBI:txid243277); Van, *Vibrio anguillarum* (NCBI:txid882102); Vfu, *Vibrio furnissii* (NCBI:txid903510); Vha, *Vibrio harveyi* (NCBI:txid338187); Vpa, *Vibrio parahaemolyticus* (NCBI:txid223926); Val, *Vibrio alginolyticus* (NCBI:txid1219076); Vtu, *Vibrio tubiashii* (NCBI:txid1051646).

Figure S6: Expression profiles of selected QS genes after autoinducer treatment. A *V. cholerae luxS*/*cqsA*/*tdh* triple mutant was cultivated in the absence (-) or presence (+) of autoinducers (5 µM final conc. each) in M9 minimal media. RNA was collected at OD_{600}=0.2 and sequenced (Dataset S1). Bars represent the normalized expression values of the indicated genes, relative to mock treated cells (set to 1). Black bars represent the expression values of the second gene of the respective operon. Error bars indicate the SD of three biological replicates. The charts are clustered by transcripts coding for receptor proteins (A), proteins involved in signal transduction (B) and transcriptional regulators (C).

Figure S7: MCPs and c-di-GMP related genes that are differentally expressed in response to AI-2, CAI-1 and DPO. Heatmaps illustrate profiles of transcripts coding for methyl-accepting chemotaxis proteins (A) and mRNAs linked to c-di-GMP production, degradation or binding (B) that were differentally expressed in the autoinducer RNA-seq experiment (Dataset S1/Table S1; experimental procedure described in the text and in Fig S4).

Fold changes of the normalized expression values were calculated relative to the normalized expression values of the mock treated replicates and log2 transformed.

Supplementary Materials and Methods

Plasmid construction

All plasmids used in this study are listed in Table S3. Plasmid pCMW-1C was obtained by Gibson Assembly (GA) (2) of linearized pCMW-1 (pCtr), using KPO-1520/1521, and the Cm^R cassette, which was amplified from pCP20 with KPO-1518/1519. The *gfp* in the pCMW-1C plasmid was replaced with the *mKate2* gene, which was amplified with KPO-1736/1737. GA was performed after the plasmid was linearized using KPO-1734/1735. The obtained plasmid was pYH-010 and served as backbone for the transcrictional reporter plasmids pMD-079, pMD-156-169, and pRH-069. To generate these reporters, the pYH-010 plasmid and the promoter inserts were digested with SphI and SalI restriction enzymes and ligated with T4 ligase. Promoter regions of *ctxA*, *tcpP*, *toxR*, *toxT*, *tcpA* and *aphB* were amplified from KPS-0014 genomic DNA (gDNA) using the following sets of oligonucleotides: KPO-2222/2223, KPO-2595/2596, KPO-2597/2598, KPO-2599/2600, KPO-2601/2602, and KPO-2591/2592. The plasmids pRH-030 and pRH-062 were constructed by GA, using the pKAS32 backbone that was linearized with KPO-0267/0268. The insert fragments for pRH-030 were amplified from KPS-0014 gDNA (KPO-2349/2350 and KPO-2353/2354) and from KPS-0518 (KPO-2351/2352) gDNA that carried the 3XFLAG coding sequence. Both insert fragments for pRH-062 were obtained from KPS-0014 gDNA using KPO-2931/2932 and KPO-2933/2934, respectively. pKP-333 served as template for plasmids pRH-031-036 using oligonucleotides combinations KPO-0092/2171, KPO-0092/2172, KPO-0092/2173, KPO-2535/2536, KPO-2535/2537, and KPO-2535/2538, respectively. The linearized products were ligated following the GA protocol. The pNP-002 plasmid was generated by XbaI digest and T4 ligation of the pEVS143 plasmid and the *vcr089* insert, that was amplified from KPS-0014 gDNA using KPO-0999/1000. Plasmids pRH-037 and pRH-038 were constructed by GA and used linearized pKP-333 (KPO-0631/2539) and pNP-002 (KPO-0631/2542) as backbones, respectively. The terminator insert fragments derived from the same template plasmids, however, in a reciprocal way. The *vcr089* terminator for pRH-037 was amplified from pNP-002 using KPO-2540/2541, whereas the *vqmR* terminator sequence for pRH-038 was obtained from pKP-333 using the oligonucleotides KPO-2543/2544. Translational *gfp* reporter fusions were designed as previously described (3) and oligonucleotides for cloning are listed in Table S4. Single point mutation in the *vqmR* gene on plasmids pRH-063 was introduced by site-directed mutagenesis using template plasmid pKP-333 and primer set KPO-2709/2710. The plasmid pKP-462 served as template DNA for single point mutations in the *aphA* 5' UTR of plasmids pRH-064, pRH-072, pRH-073 and pRH-074 using site-directed mutagenesis and primer sets KPO-2711/2712, KPO-4186/4190, KPO-4187/4191 and KPO-4188/4192, respectively.

Construction of *V. cholerae* mutant strains

A list of all strains used in this study is provided in Table S2. Throughout the study *V. cholerae* C6706 was used as the wild-type strain. All *V. cholerae* mutants were generated using the pKAS32 suicide vector (4) and established cloning strategies (5). Briefly, pKAS32 plasmids (pKAS32-cqsA, pRH-030, and pRH-062) were transferred into *V. cholerae* strains by conjugation and cells were screened for Ampicillin resistance. Single colonies were streaked on Streptomycin plates and colonies were tested for desired mutations by PCR. The KPEC-50812 strain was generated from KFS-0028 using the pCP20 plasmid as described previously (6).

qRT-PCR

Quantitative real-time PCR was performed as described previously (7). Briefly, total RNA was isolated using the SV Total RNA Isolation System (Promega) according to manufacterer's instructions. Novel VqmR targets were tested by qRT-PCR in a MyiQ™ Single-Color Real-Time PCR Detection System (Bio-Rad) with *recA* as reference gene. Oligonucleotides used in qRT-PCR experiments are listed in Table S4.

T7 transcription and 5' end labelling of RNA

A DNA template carrying a T7 promoter sequence for *in vitro* transcription of *aphA* mRNA (-204 to +60 relative to the translational start site) was generated by PCR (KPO-4075/4077). RNA was transcribed using the AmpliScribe T7-Flash Transcription kit (Lucigen) according to the manufacturer's recommendations. Size and integrity of the RNA were confirmed on a denaturing polyacrylamide gel, and 5'-end labelling was performed as described previously (7).

Toeprinting analysis

In vitro 30S toeprint experiments were carried out as described previously (8) with few modifications. For annealing of the primer, an unlabelled *aphA* mRNA fragment (0.2 pmol) and 0.5 pmol 5' end-labelled oligo (KPO-4077) were denatured together and adjacently chilled on ice for 5 min. For inhibition analysis, 0.2, 2 or 10 pmol of an LNA mimicking the VqmR interaction on *aphA* mRNA (KFO-0859) was added, and incubated at 37°C for 10 min. Samples were mixed with 2 pmol purified 30S ribosomal subunit, and 10 pmol uncharged tRNA-fMet. Reverse transcription was initiated by addition of 100 U SuperScriptII (Invitrogen). Following cDNA synthesis, template RNA was digested by alkaline hydrolysis, and cDNA was precipitated. Samples were separated in the presence of a gene specific sequencing ladder by denaturing PAGE on 6% sequencing gels at constant power of 30 W. The sequencing ladder was generated using the Sequenase Cycle Sequencing Kit (Thermo; 785001KT)

following the manufacturer’s instructions using the same PCR-generated DNA template as in RNA synthesis and the same 5′ end-labeled primer as in toeprinting reactions.

Supplemental References

1. Corpet, F. (1988) Multiple sequence alignment with hierarchical clustering. *Nucleic Acids Res*, **16**, 10881-10890.
2. Gibson, D.G. (2009) Synthesis of DNA fragments in yeast by one-step assembly of overlapping oligonucleotides. *Nucleic Acids Res*, **37**, 6984-6990.
3. Corcoran, C.P., Podkaminski, D., Papenfort, K., Urban, J.H., Hinton, J.C. and Vogel, J. (2012) Superfolder GFP reporters validate diverse new mRNA targets of the classic porin regulator, MicF RNA. *Mol Microbiol*, **84**, 428-445.
4. Skorupski, K. and Taylor, R.K. (1996) Positive selection vectors for allelic exchange. *Gene*, **169**, 47-52.
5. Drescher, K., Nadell, C.D., Stone, H.A., Wingreen, N.S. and Bassler, B.L. (2014) Solutions to the public goods dilemma in bacterial biofilms. *Curr Biol*, **24**, 50-55.
6. Datsenko, K.A. and Wanner, B.L. (2000) One-step inactivation of chromosomal genes in Escherichia coli K-12 using PCR products. *Proc Natl Acad Sci U S A*, **97**, 6640-6645.
7. Papenfort, K., Pfeiffer, V., Mika, F., Lucchini, S., Hinton, J.C. and Vogel, J. (2006) SigmaE-dependent small RNAs of Salmonella respond to membrane stress by accelerating global omp mRNA decay. *Mol Microbiol*, **62**, 1674-1688.
8. Sharma, C.M., Darfeuille, F., Plantinga, T.H. and Vogel, J. (2007) A small RNA regulates multiple ABC transporter mRNAs by targeting C/A-rich elements inside and upstream of ribosome-binding sites. *Genes Dev*, **21**, 2804-2817.
9. Thelin, K.H. and Taylor, R.K. (1996) Toxin-coregulated pilus, but not mannose-sensitive hemagglutinin, is required for colonization by Vibrio cholerae O1 El Tor biotype and O139 strains. *Infect Immun*, **64**, 2853-2856.
10. Papenfort, K., Forstner, K.U., Cong, J.P., Sharma, C.M. and Bassler, B.L. (2015) Differential RNA-seq of Vibrio cholerae identifies the VqmR small RNA as a regulator of biofilm formation. *Proc Natl Acad Sci U S A*, **112**, E766-775.
11. Papenfort, K., Silpe, J.E., Schramma, K.R., Cong, J.P., Seyedsayamdost, M.R. and Bassler, B.L. (2017) A Vibrio cholerae autoinducer-receptor pair that controls biofilm formation. *Nat Chem Biol*, **13**, 551-557.
12. de Lorenzo, V. and Timmis, K.N. (1994) Analysis and construction of stable phenotypes in gram-negative bacteria with Tn5- and Tn10-derived minitransposons. *Methods Enzymol*, **235**, 386-405.
13. Waters, C.M. and Bassler, B.L. (2006) The Vibrio harveyi quorum-sensing system uses shared regulatory components to discriminate between multiple autoinducers. *Genes Dev*, **20**, 2754-2767.
14. Antonova, E.S. and Hammer, B.K. (2011) Quorum-sensing autoinducer molecules produced by members of a multispecies biofilm promote horizontal gene transfer to Vibrio cholerae. *FEMS Microbiol Lett*, **322**, 68-76.

Figure S1

Herzog *et al.*, 2018

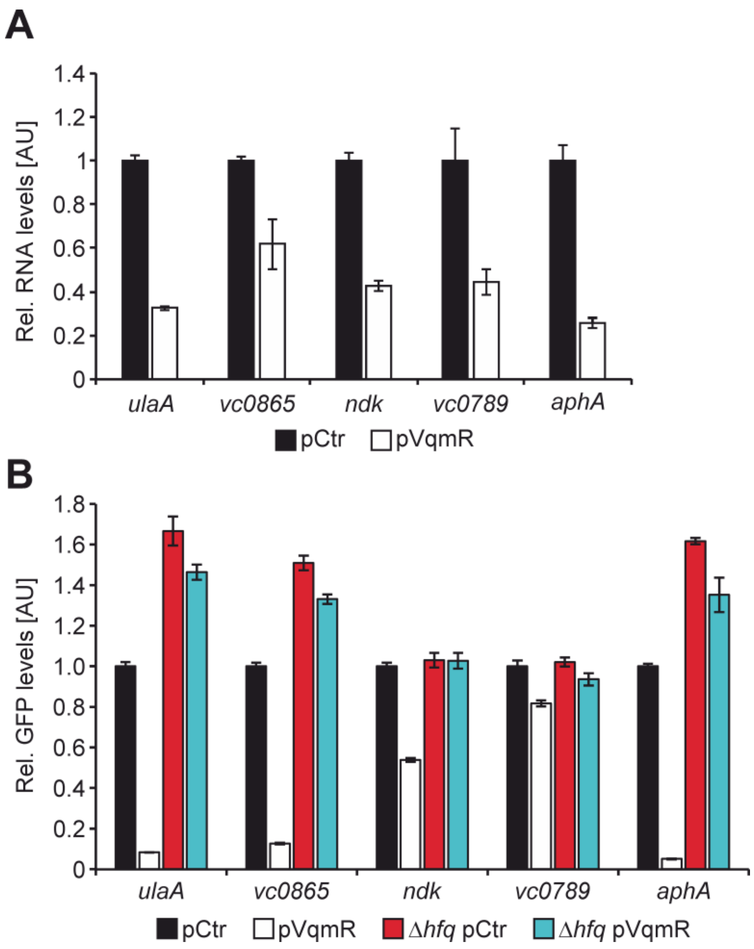

Figure S2

Herzog *et al.*, 2018

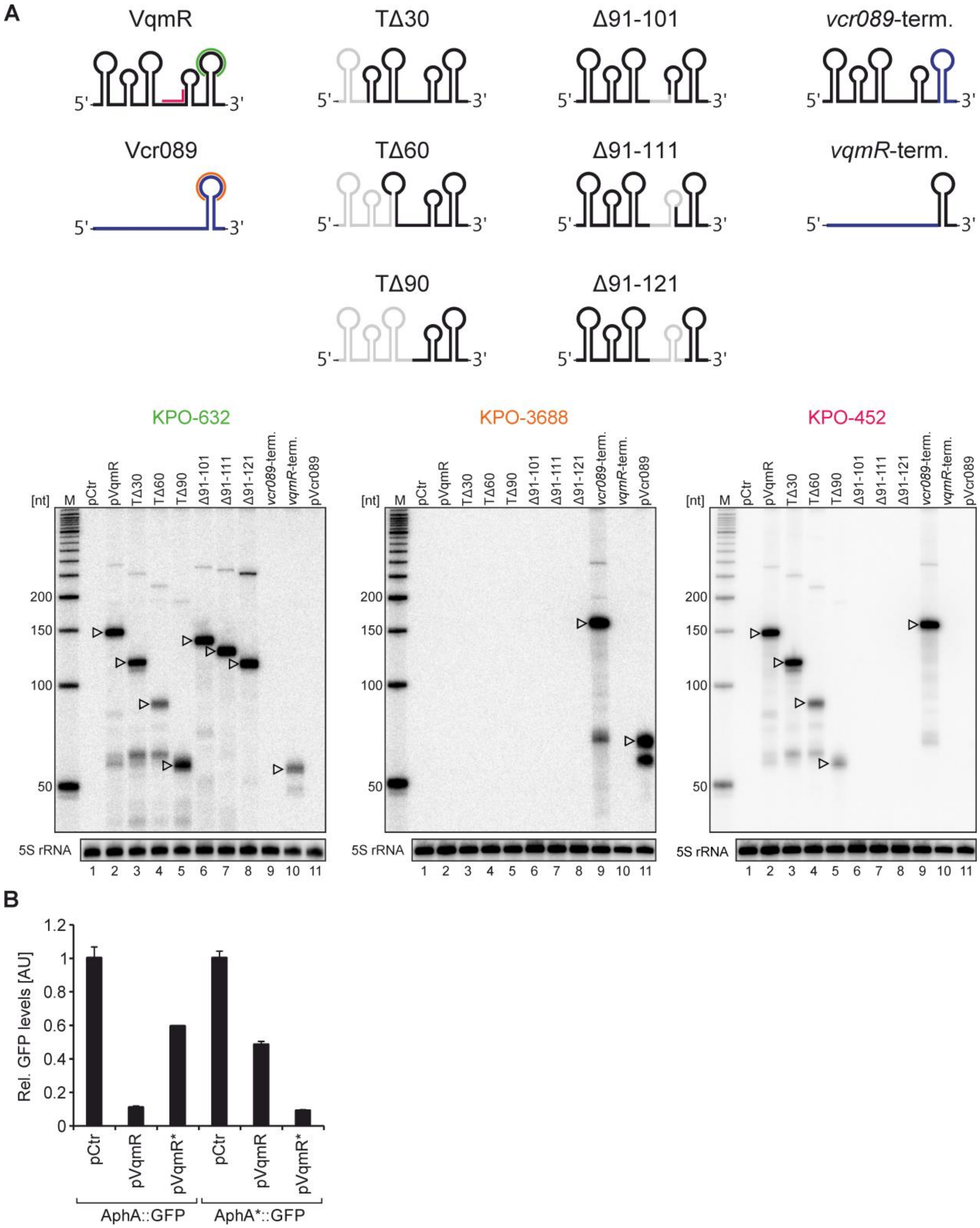

Figure S3

Herzog *et al.*, 2018

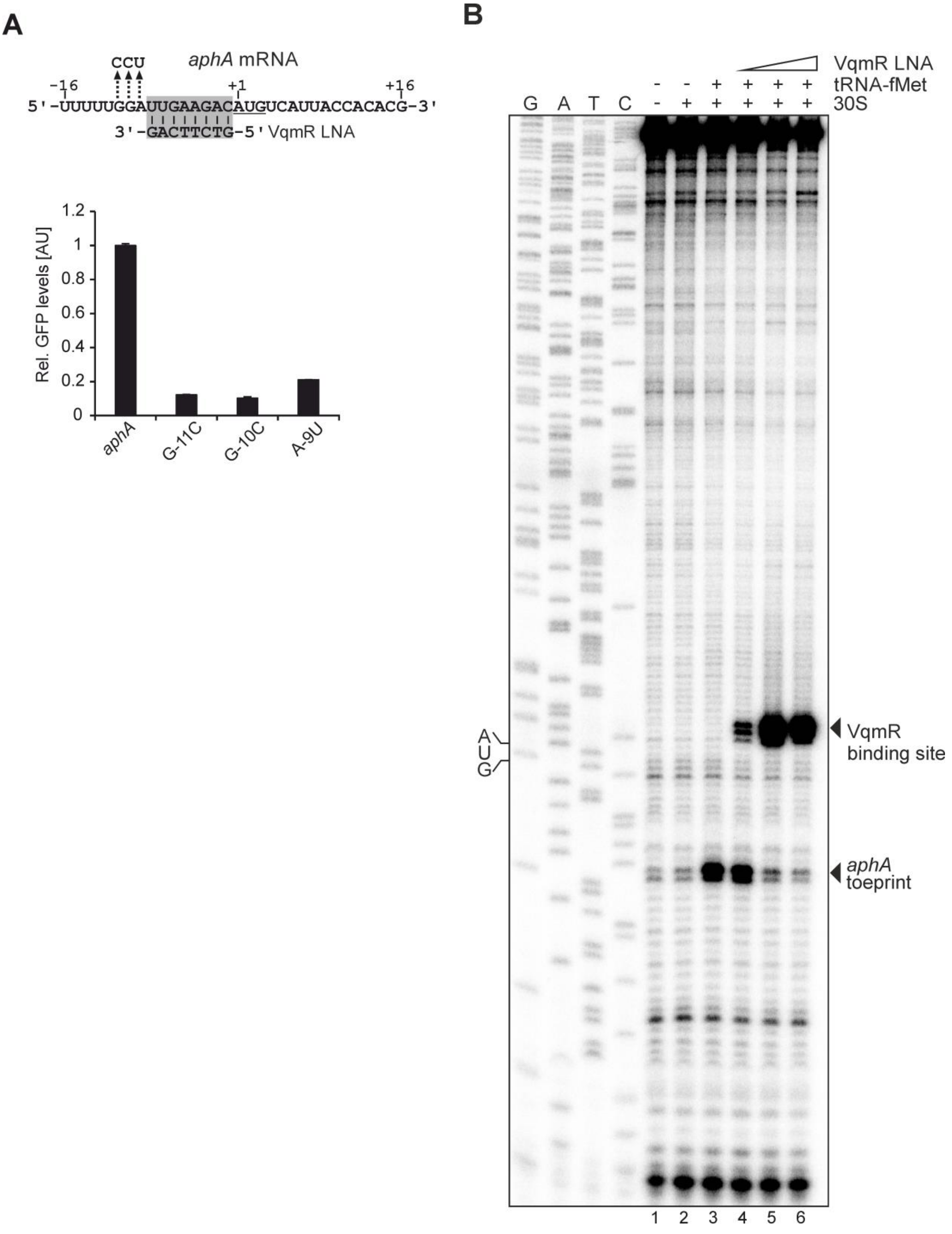

Figure S4

Herzog *et al.*, 2018

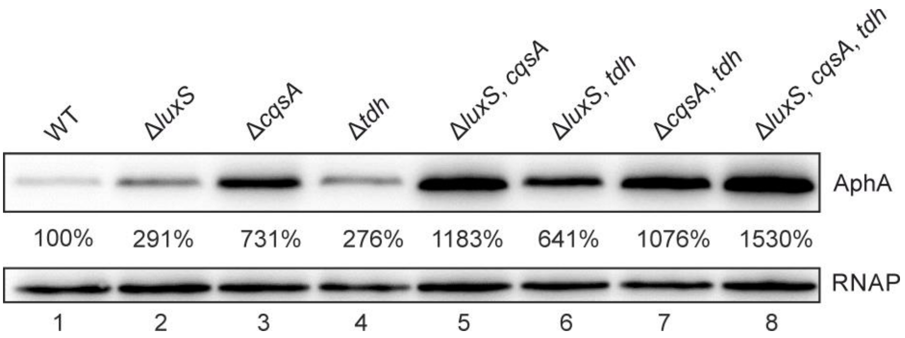

Figure S5

Herzog *et al.*, 2018

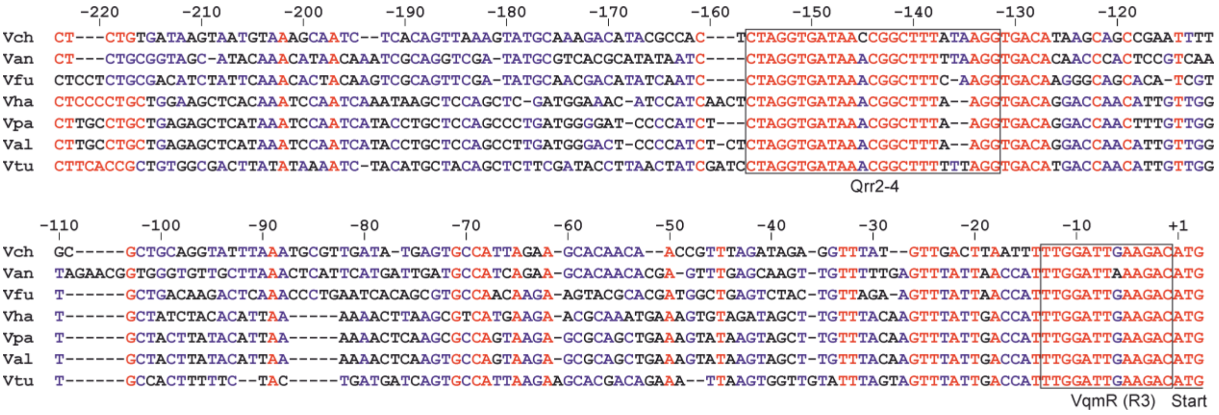

Figure S6

Herzog *et al.*, 2018

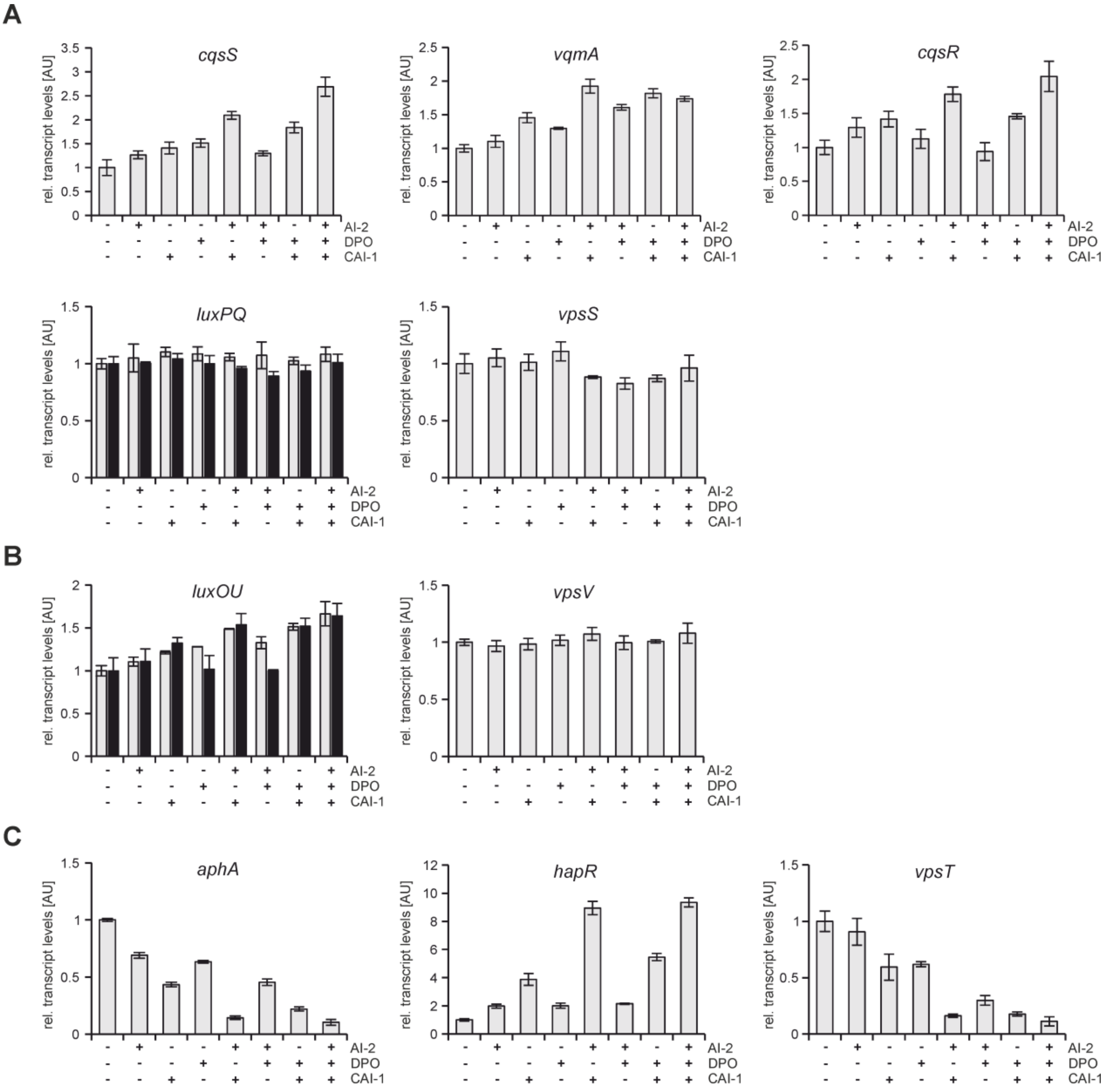

Figure S7

Herzog *et al.*, 2018

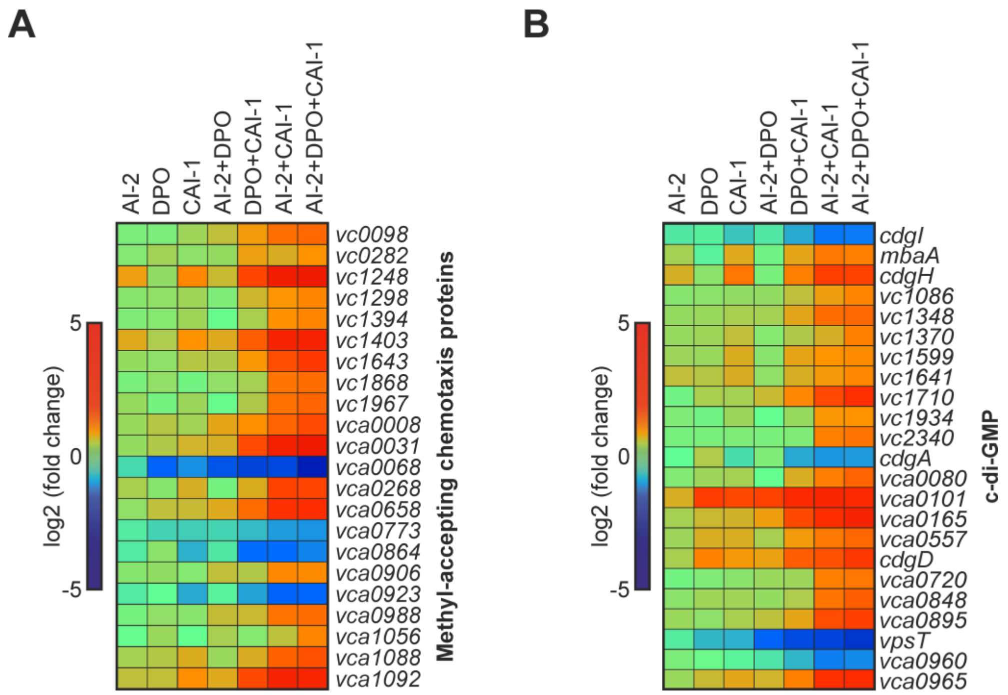

Table S2 Bacterial strains used in this study

Strain	Relevant markers/ genotype	Reference/ source
V. cholerae		
KPS-0014	Wild-type C6706	(9)
KPS-0429	Δ*vqmR* C6706	(10)
KPS-0431	Δ*vqmA* C6706	(10)
KPS-0518	*vpsT*::3XFLAG C6706	(10)
KPS-0842	Δ*tdh* C6706	(11)
KPVC-11005	*aphA*::3XFLAG C6706	This study
KPVC-11006	Δ*vqmA*/*aphA*::3XFLAG C6706	This study
KPVC-11007	Δ*vqmR*/*aphA*::3XFLAG C6706	This study
KPVC-11008	Δ*tdh*/*aphA*::3XFLAG C6706	This study
KPVC-11861	Δ*vqmR*/Δ*tdh* C6706	This study
KPVC-11263	Δ*luxS*/Δ*cqsA*/Δ*tdh* C6706	This study
KPVC-11264	Δ*luxS*/*aphA*::3XFLAG C6706	This study
KPVC-11265	Δ*cqsA*/*aphA*::3XFLAG C6706	This study
KPVC-11266	Δ*luxS*/Δ*cqsA*/*aphA*::3XFLAG C6706	This study
KPVC-11267	Δ*luxS*/Δ*tdh*/*aphA*::3XFLAG C6706	This study
KPVC-11268	Δ*cqsA*/Δ*tdh*/*aphA*::3XFLAG C6706	This study
KPVC-11269	Δ*luxS*/Δ*cqsA*/Δ*tdh*/*aphA*::3XFLAG C6706	This study
E. coli		
TOP10	*mcr*AΔ*(mrr-hsd*RMS-*mcr*BC) Φ80*lac*ZΔM15Δ*lac*X74*deo*R*rec*A1 *ara*D139Δ*(ara-leu*)7697 *gal*U *gal*K *rps*L *end*A1 *nup*G	Invitrogen
S17λpir	Δ*lacU*169 (Φ*lacZ*ΔM15), *recA*1, *endA*1, *hsdR*17, *thi*-1, *gyrA*96, *relA*1, λ*pir*	(12)
KFS-0028	*hfq::kanR* MC4100	Laboratory stock
KPEC-50812	Δ*hfq* MC4100	This study

Table S3 Plasmids used in this study

Plasmid trivial name	Plasmid stock name-	Relevant fragment	Comment	Origin, marker	Reference
pBAD	pBAD		Control plasmid	P15A, Kan^R	(10)
pCtr	pCMW-1		Control plasmid	P15A, Kan^R	(13)
pCMW-1C	pCMW-1C	Cm^R cassette	Promoterless plasmid for transcriptional reporters	P15A, Cm^R	Papenfort plasmid collection
pCMW-1C-mKate2	pYH-010	mKate2	Promoterless plasmid for transcriptional reporters	P15A, Cm^R	Papenfort plasmid collection
pCP20	pCP20	FLP recombinase	Plasmid to cure mutants with antibiotic markers	PSC101*, Cm^R	(6)
pEVS143	pEVS143	Ptac promoter	Constitutive over-expression plasmid	P15A, KanR	(10)
pKAS32	pKAS32		suicide plasmid for allelic exchange	R6K, Amp^R	(4)
pKAS32-*cqsA*	pKAS32-cqsA	up-/downstream flanks of *cqsA*	suicide plasmid for *cqsA* knock-out	R6K, Amp^R	(14)
pBAD-*vqmR*	pKP-331	VqmR	pBAD-*vqmR* expression plasmid	P15A, Kan^R	(10)
pVqmR	pKP-333	VqmR	VqmR expression plasmid	P15A, Kan^R	(9)
pVqmRΔR1	pKP-344	VqmRΔR1	VqmRΔR1 expression plasmid	P15A, Kan^R	(5)
pVqmRΔR2	pKP-345	VqmRΔR2	VqmRΔR2 expression plasmid	P15A, Kan^R	(10)
p*aphA::gfp*	pKP-462	*aphA::gfp*	translational reporter *aphA::gfp*	PSC101*, Cm^R	Papenfort plasmid collection
p*ctxAB::mKate2*	pMD-079	P*ctxAB::mKate2*	transcriptional reporter *ctxAB*	P15A, Cm^R	Papenfort plasmid collection
p*tcpPH::mKate2*	pMD-156	P*tcpPH::mKate2*	transcriptional reporter *tcpPH*	P15A, Cm^R	Papenfort plasmid collection
p*toxRS::mKate2*	pMD-157	P*toxRS::mKate2*	transcriptional reporter *toxRS*	P15A, Cm^R	Papenfort plasmid collection
p*toxT::mKate2*	pMD-158	P*toxT::mKate2*	transcriptional reporter *toxT*	P15A, Cm^R	Papenfort plasmid collection
p*tcpA-F::mKate2*	pMD-159	P*tcpA-F::mKate2*	transcriptional reporter *tcpA-F*	P15A, Cm^R	Papenfort plasmid collection
pVcr089	pNP-002	Vcr089	Vcr089 expression plasmid	P15A, Kan^R	Papenfort plasmid collection
pKAS32-*aphA::3XFLAG*	pRH-030	*3XFLAG* sequence	*aphA::3XFLAG* allelic replacement	R6K, Amp^R	This study
pVqmR (121nt)	pRH-031	VqmR (121 nt)	VqmR (121nt) expression plasmid	P15A, Kan^R	This study
pVqmR (91nt)	pRH-032	VqmR (91nt)	VqmR (91nt) expression plasmid	P15A, Kan^R	This study
pVqmR (61nt)	pRH-033	VqmR (61nt)	VqmR (61nt) expression plasmid	P15A, Kan^R	This study

pVqmR (Δ91-101nt)	pRH-034	VqmR (Δ91-101nt)	VqmR (Δ91-101nt) expression plasmid	P15A, Kan^R	This study
pVqmR (Δ91-111nt)	pRH-035	VqmR (Δ91-111nt)	VqmR (Δ91-111nt) expression plasmid	P15A, Kan^R	This study
pVqmR (Δ91-121nt)	pRH-036	VqmR (Δ91-121nt)	VqmR (Δ91-121nt) expression plasmid	P15A, Kan^R	This study
pVqmR-*vcr089*-term.	pRH-037	VqmR-*vcr089*-terminator	VqmR-*vcr089*-term. expression plasmid	P15A, Kan^R	This study
pVrc089-*vqmR*-term.	pRH-038	Vrc089-*vqmR*-terminator	Vrc089-*vqmR*-term. expression plasmid	P15A, Kan^R	This study
pKAS32-*luxS*	pRH-062	up-/downstream flanks of *luxS*	suicide plasmid for *luxS* knock-out	R6K, Amp^R	This study
pVqmR (C133G)	pRH-063	VqmR (C133G)	VqmR (C133G) expression plasmid	P15A, Kan^R	This study
p*aphA::gfp* (G-3C)	pRH-064	*aphA::gfp* (G-3C)	translational reporter *aphA::gfp* (G-3C)	PSC101*, Cm^R	This study
p*vc0865::gfp*	pRH-065	*vc0865::gfp*	translational reporter *vc0865::gfp*	PSC101*, Cm^R	This study
p*ndk::gfp*	pRH-066	*ndk::gfp*	translational reporter *ndk::gfp*	PSC101*, Cm^R	This study
p*ulaA::gfp*	pRH-067	*ulaA::gfp*	translational reporter *ulaA::gfp*	PSC101*, Cm^R	This study
p*vc0789::gfp*	pRH-068	*vc0789::gfp*	translational reporter *vc0789::gfp*	PSC101*, Cm^R	This study
p*aphB::mKate2*	pRH-069	P*aphB::mKate2*	transcriptional reporter *aphB*	P15A, Cm^R	This study
p*aphA::gfp* (G-11C)	pRH-072	*aphA::gfp* (G-11C)	translational reporter *aphA::gfp* (G-11C)	PSC101*, Cm^R	This study
p*aphA::gfp* (G-10C)	pRH-073	*aphA::gfp* (G-10C)	translational reporter *aphA::gfp* (G-10C)	PSC101*, Cm^R	This study
p*aphA::gfp* (A-9U)	pRH-074	*aphA::gfp* (A-9U)	translational reporter *aphA::gfp* (A-9U)	PSC101*, Cm^R	This study
pXG10-SF	pXG10SF	'*lacZ::gfp*	template plasmid for translational reporters	PSC101*, Cm^R	(3)

Table S4 DNA oligonucleotides used in this study

Sequences are given in 5' → 3' direction; 5' P denotes a 5' monophosphate

ID	Sequence	Description
KFO-0859	GTCTTCAG	VqmR LNA
KPO-0063	CGTCTATAAGTGTGAACAATGGTG	Qrr4 oligoprobe
KPO-0092	CCACACATTATACGAGCCGA	pRH-031-033
KPO-0243	TTCGTTTCACTTCTGAGTTCGG	5S rRNA oligoprobe
KPO-0267	TAATAGGCCTAGGATGCATATG	pRH-030/062
KPO-0268	CGTTAACAACCGGTACCTCTA	pRH-030/062
KPO-0452	ATCTTGTCGACGTGTAGAAGAGGTT	VqmR oligoprobe
KPO-0631	P-CATCCAAACAGTCCCCGAAT	pRH-037/038
KPO-0632	GCCAGCCTGAAGACGGGCT	VqmR oligoprobe
KPO-0999	P-ACCACTGCTTTTTCTTAGAAGAC	pNP-002
KPO-1000	GTTTTTTCTAGAGGATTAGAACCCGAATTAAACT	pNP-002
KPO-1058	GTTTTTTATGCATCTCTGTGATAAGTAATGTAAAGC	pKP-462
KPO-1059	GTTTTTTGCTAGCATCGTACCCGGTTGCATCG	pKP-462
KPO-1518	CAGGATTGTGTAGGCTGG	pCMW-1C
KPO-1519	CCTCCTTAGTTCCTATTCC	pCMW-1C
KPO-1520	CCAGCCTACACAATCCTGAGCACCGCCAGGTGCG	pCMW-1C
KPO-1521	GGAATAGGAACTAAGGAGGGGCAAAGCCGTTTTTCCATAG	pCMW-1C
KPO-1702	ATGCATGTGCTCAGTATCTCTATC	pRH-065-068
KPO-1703	GCTAGCGGATCCGCTGG	pRH-065-068
KPO-1734	TGAGGATCCGGTGATTGATT	pYH-010
KPO-1735	AACTAGGCCTGTCGACAGAT	pYH-010
KPO-1736	ATCTGTCGACAGGCCTAGTTGCGTACAGGAGGTGTGAAATG	pYH-010
KPO-1738	AATCAATCACCGGATCCTCAAAAAAAAAGCACCCCGGTTTG	pYH-010
KPO-2171	TCGGCTCGTATAATGTGTGGTATCAATATGATACCTCTGTGTG	pRH-031
KPO-2172	TCGGCTCGTATAATGTGTGGGACCCTCGCTGGATTCATG	pRH-032
KPO-2173	TCGGCTCGTATAATGTGTGGACACGTCGACAAGATATGTTTC	pRH-033
KPO-2211	TTCAGTACAATCGGCAGAAC	*aphA* qRT-PCR
KPO-2212	GGCTGGGTTAGCGTAATAAG	*aphA* qRT-PCR
KPO-2222	GTTTTTGCATGCGTGTTCGATACCTTTGCAGC	pMD-079
KPO-2223	GTTTTTGTCGACAGAAAAATAATTGATCAAAACAATT	pMD-079
KPO-2236	GTTGACTTAATTTTTGGATTGAAGAC	*aphA* riboprobe
KPO-2237	GTTTTTTTAATACGACTCACTATAGGGAGGCAGAAGTAGCCAATGCTAGCA	*aphA* riboprobe
KPO-2349	AGAGGTACCGGTTGTTAACGGTCACAACTTTGTGGCCTTTTG	pRH-030
KPO-2350	TGCCATCGCGTTCAATTCTG	pRH-030
KPO-2351	CAGAATTGAACGCGATGGCAGACTACAAAGACCATGACGGT	pRH-030
KPO-2352	ATCGACAGGTTTGGCTTGGCTTACTATTTATCGTCATCTTTGTAGTC	pRH-030
KPO-2353	GCCAAGCCAAACCTGTCGAT	pRH-030
KPO-2354	TATGCATCCTAGGCCTATTACGGCCTTGAGCTATATTCTG	pRH-030
KPO-2378	GGTAACCCAGAAACTACCACTG	*recA* qRT-PCR
KPO-2379	CACCACTTCTTCGCCTTCTT	*recA* qRT-PCR
KPO-2535	AGAAGAGGTTCCATGAATCCAG	pRH-034-036
KPO-2536	GGATTCATGGAACCTCTTCTAAGATATGTTTCTTGTATTTATGCC	pRH-034
KPO-2537	GGATTCATGGAACCTCTTCTTCTTGTATTTATGCCAGCCCG	pRH-035
KPO-2538	GGATTCATGGAACCTCTTCTATGCCAGCCCGTCTTCAG	pRH-036
KPO-2539	ATAAATACAAGAAACATATCTTGTCG	pRH-037
KPO-2540	GATATGTTTCTTGTATTTATGGCCCTAAATCTGTACTAGG	pRH-037
KPO-2541	ATTCGGGGACTGTTTGGATGAAGAAAAAAGCCCTAAACCTAGTAC	pRH-037
KPO-2542	TTCTTAGAGTCTTCTAAGAAAAAGC	pRH-038

KPO-2543	TTCTTAGAAGACTCTAAGAAGCC AGCCCGTCTTCAGG	pRH-038
KPO-2544	ATTCGGGGACTGTTTGGATGAAAAAAAGCCAGCCTGAAGACG	pRH-038
KPO-2591	GTCGACAGGCCTAGTTG	pRH-069
KPO-2592	GCATGCAAAAAGACCCTTC	pRH-069
KPO-2595	GAAGGGTCTTTTTGCATGCGTCACAGGAAAGATAATGTAAC	pMD-156
KPO-2596	CAACTAGGCCTGTCGACCATTTTCTTAATAATAACTACCC	pMD-156
KPO-2597	GAAGGGTCTTTTTGCATGCTTGGAGTACAAAGAGTGAATC	pMD-157
KPO-2598	CAACTAGGCCTGTCGACAAATGCAGACTTAGAATAATCC	pMD-157
KPO-2599	GAAGGGTCTTTTTGCATGCGGTCAAATACTATGTTCTCAAATTC	pMD-158
KPO-2600	CAACTAGGCCTGTCGACATCGAAGTTAATATAAAACTACATAAC	pMD-158
KPO-2601	GAAGGGTCTTTTTGCATGCACCCATTTTTTATCGTCATTCAT	pMD-159
KPO-2602	CAACTAGGCCTGTCGACAGCAATACGCACATTTAACC	pMD-159
KPO-2709	CCGTGTTCAGGCTGGCTTTTTTTC	pRH-063
KPO-2710	CTGAACACGGGCTGGCATAAATAC	pRH-063
KPO-2711	GAACACATGTCATTACCACACGTTATC	pRH-064
KPO-2712	GACATGTGTTCAATCCAAAAATTAAGTCAAC	pRH-064
KPO-2812	TCTTGCTATTGGCCAGCATAC	*vc0865* qRT-PCR
KPO-2813	GGTTCCCTGACGTTAAGTAGTC	*vc0865* qRT-PCR
KPO-2814	TGACTTCTTCGGTCGGTTTG	*ulaA* qRT-PCR
KPO-2815	CCATCATTTATTCGGCGGTATTG	*ulaA* qRT-PCR
KPO-2828	GATCATGGTGCAGGTCTTAGAG	*ndk* qRT-PCR
KPO-2829	GCGGCTTCTTCTGGGTTAG	*ndk* qRT-PCR
KPO-2931	AGAGGTACCGGTTGTTAACGCGTAATTACTACCGTTTTGGTTG	pRH-062
KPO-2932	AACAAAAGCCCGAACTCAACCTAAAACTAAATTAACTCACCGCC	pRH-062
KPO-2933	GTTGAGTTCGGGCTTTTGTTTG	pRH-062
KPO-2934	TATGCATCCTAGGCCTATTACTGGTGTTTGCCGAAGTCAC	pRH-062
KPO-3288	GCGCTGAGATTAACTGCTTAAC	*vc0789* qRT-PCR
KPO-3289	CTGACAAAGTGGGATCACAAATAC	*vc0789* qRT-PCR
KPO-3290	GAGATACTGAGCACATGCATGGGCGACTCAATAGCCACAG	pRH-068
KPO-3291	GAGCCAGCGGATCCGCTAGCCAACCAATTTTCTCGCGCTTGG	pRH-068
KPO-3292	GAGATACTGAGCACATGCATCAATAATAAAGCAGATCGATAAGCG	pRH-065
KPO-3293	GAGCCAGCGGATCCGCTAGCTCTGCGGTTAGCGGATACGG	pRH-065
KPO-3294	GAGATACTGAGCACATGCATTACCCTTCCAATTTGACGCTGC	pRH-066
KPO-3295	GAGCCAGCGGATCCGCTAGCAATCAAGTTACGCTTCACCGCA	pRH-066
KPO-3296	GAGATACTGAGCACATGCATGTGAGCAAAAGGAGCGATTTTAC	pRH-067
KPO-3297	GAGCCAGCGGATCCGCTAGCAGGGGCTTTGGTCATTACTTG	pRH-067
KPO-3566	TGAAGGGTCTTTTTGCATGCCCGCGAACTGGGCATTACAC	pRH-069
KPO-3567	ACGCAACTAGGCCTGTCGACACACATATAAGCCGATAAATTTTGCG	pRH-069
KPO-3688	CCCTAAACCTAGTACAGATTTAG	Vcr089 oligoprobe
KPO-4075	GTTTTTTTTAATACGACTCACTATAGCTCTGTGATAAGTAATGTAAAGCAA	*aphA* T7 transcription
KPO-4077	ATCGTACCCGGTTGCATCG	*aphA* T7 transcription
KPO-4186	CTTAATTTTTCGATTGAAGACATGTCATTACC	pRH-072
KPO-4187	CTTAATTTTTGCATTGAAGACATGTCATTACC	pRH-073
KPO-4188	CTTAATTTTTGGTTTGAAGACATGTCATTACC	pRH-074
KPO-4190	GTCTTCAATCGAAAAATTAAGTCAACATAAACC	pRH-072
KPO-4191	GTCTTCAATGCAAAAATTAAGTCAACATAAACC	pRH-073
KPO-4192	GTCTTCAAACCAAAAATTAAGTCAACATAAACC	pRH-074
